# Harnessing multi-omics and artificial intelligence: revolutionizing prognosis and treatment in hepatocellular carcinoma

**DOI:** 10.3389/fimmu.2025.1592259

**Published:** 2025-07-23

**Authors:** Zhen Wang, Gangchen Zhou, Rongchuan Cao, Guolin Zhang, Yongxu Zhang, Mingyue Xiao, Longbi Liu, Xuesong Zhang

**Affiliations:** ^1^ Department of Interventional Therapy, Zuanshiwan Campus, The Second Hospital of Dalian Medical University, Dalian, Liaoning, China; ^2^ Department of Graduate, Dalian Medical University, Dalian, Liaoning, China; ^3^ Department of General Surgery, The First Affiliated Hospital of Dalian Medical University, Dalian, Liaoning, China; ^4^ Department of Orthopedics, The Second Hospital of Dalian Medical University, Dalian, Liaoning, China; ^5^ Department of Cardiology II, The Second Hospital of Dalian Medical University, Dalian, Liaoning, China

**Keywords:** hepatocellular carcinoma (HCC), multi-omics, artificial intelligence-derived risk score (AIDRS), molecular subtypes, sorafenib, transcatheter arterial chemoembolization (TACE), immunotherapy, CEP55

## Abstract

**Background:**

Hepatocellular carcinoma (HCC) is the most prevalent form of liver cancer, characterized by elevated mortality rates and heterogeneity. Despite advancements in treatment, the development of personalized therapeutic strategies for HCC remains a substantial challenge due to the intricate molecular characteristics of the disease. A multi-omics approach has the potential to offer more profound insights into HCC subtypes and enhance patient stratification for personalized treatments.

**Methods:**

A comprehensive data set comprising clinical, transcriptomic, genomic and epigenomic information from HCC patients was retrieved from the TCGA, ICGC, GEO and CPTAC databases. To identify distinct molecular subtypes, a multi-omics data integration approach was employed, utilizing 10 distinct clustering algorithms. Survival analysis, immune infiltration profiling and drug sensitivity predictions were then used to evaluate the prognostic significance and therapeutic responses of these subtypes. Furthermore, machine learning models were employed to develop the artificial intelligence-derived risk score (AIDRS) with the aim of predicting patient outcomes and guiding personalized therapy. *In vitro* and vivo experiments were conducted to assess the role of CEP55 in tumor progression.

**Results:**

The present study identified two distinct HCC subtypes (CS1 and CS2, respectively), each exhibiting different clinical outcomes and molecular characteristics. CS1 was associated with better overall survival, while CS2 exhibited higher mutation burden and immune suppression. The AIDRS, constructed using a multi-step machine learning approach, effectively predicted patient prognosis across multiple cohorts. High AIDRS score correlated with poor prognosis and a limited response to immunotherapy. Furthermore, the study identified CEP55 as a potential therapeutic target, as it was found to be overexpressed in CS2 and associated with poorer outcomes. *In vitro* experiments confirmed that CEP55 knockdown reduced HCC cell proliferation, migration, and invasion. Moreover, in xenograft models, CEP55 knockdown significantly reduced tumor growth and proliferation.

**Conclusions:**

The integration of multi-omics data has been demonstrated to provide a comprehensive understanding of HCC subtypes, thus enhancing the prediction of prognosis and guiding personalized treatment strategies. The development of the AIDRS offers a robust tool for risk stratification, while CEP55 has emerged as a promising target for therapeutic intervention in HCC.

## 1 Introduction

Primary liver cancer is the sixth most prevalent form of cancer worldwide and the third leading cause of cancer-related fatalities. Hepatocellular carcinoma (HCC) accounts for approximately 75% to 85% of liver cancer cases (1). According to global cancer statistics in 2022 (2), the incidence of HCC is highest in East Asia and sub-Saharan Africa, particularly in countries like China, Japan and Mongolia. The major risk factors for HCC include chronic hepatitis B and C infections, excessive alcohol consumption and non-alcoholic fatty liver disease (NAFLD). Surgical resection is regarded as the optimal treatment option for HCC, given its status as a radical therapy. However, the majority of patients present with late-stage disease, by which point the opportunity for surgical intervention has often been missed, and the recurrence rate after surgery remains high. It is evident that local treatments such as transcatheter arterial chemoembolization (TACE) and systemic treatments, including radiotherapy, chemotherapy and immunotherapy, have become significant treatment options for HCC (1, 3, 4). Among these, sorafenib, a multi-target tyrosine kinase inhibitor, is the first targeted chemotherapeutic drug to be approved for the treatment of HCC. Although it has been shown to prolong patient survival, its efficacy is limited and drug resistance is also a prominent problem (5–7).

Recent years have seen a shift towards immunotherapy and combination targeted therapies as the prevailing trend in the treatment of HCC (1, 8, 9). A notable development is the combination of the anti- VEGFA monoclonal antibody bevacizumab and the PD-L1 inhibitor atezolizumab, which has emerged as the first treatment regimen to demonstrate a significant improvement in overall survival (OS) when compared to sorafenib (10). Furthermore, targeted drugs like lenvatinib and PD-1/PD-L1 inhibitors such as pembrolizumab have exhibited promising results (11, 12). However, systemic therapy is usually accompanied by adverse effects on normal hepatocytes, and the survival time and quality of life of patients is often seriously affected by side effects, mainly vomiting and immunosuppression (11–14). Therefore, the selection and implementation of personalized treatment regimens for HCC patients is a key challenge that needs to be addressed.

HCC is characterized by significant heterogeneity, which poses a substantial challenge to its treatment. However, this heterogeneity also presents opportunities for the development of personalized treatment strategies (1, 13). The molecular heterogeneity of HCC patients can be categorized into distinct subtypes, each with unique biological characteristics, prognosis and response to treatment (15). Elucidation of these subtypes facilitates the development of more precise and personalized treatment strategies, thereby enhancing treatment efficacy and reducing unnecessary side effects. The advent of high-throughput sequencing technology has been instrumental in facilitating the analysis of molecular subtypes of HCC, with it offering significant contributions to the prognosis, prediction and precision treatment of HCC patients (16–18). However, the majority of current research is confined to the utilization of single omics methods such as transcriptomics (19), proteomics (20) and metabolomics (21), or analysis is restricted to specific biological pathways, such as fatty acid metabolism (21). There is a paucity of systematic subtype analysis incorporating multi-omics perspectives, including genomics, transcriptomics and epigenomics, and multiple biological levels. This has impeded our ability to fully elucidate the complex biological characteristics and clinical behavior of HCC and hindered the development of more accurate predictive tools, new classification standards and biomarkers to guide individualized treatment of HCC.

In this study, we integrated multi-omics data, incorporating genomics, transcriptomics and epigenomics, to distinguish stable HCC subtypes and conduct an in-depth molecular characterization. Utilizing multiple machine learning techniques, we developed more accurate prognostic prediction models and artificial intelligence-derived risk score (AIDRS), which provide targeted guidance for specific treatment strategies for patients. This approach will contribute to the establishment of a more comprehensive and accurate personalized therapeutic strategy for HCC, ultimately improving treatment outcomes and quality of life for HCC patients.

## 2 Materials and methods

### 2.1 Multi-omics data collection and pre-processing

Clinical details, transcriptome expression (FPKM format), DNA methylation (methylation 450k format), somatic mutations (masked format) and copy number variants (gistic2 format) from TCGA-LIHC in The Cancer Genome Atlas (TCGA) database (https://portal.gdc.cancer.gov/) were downloaded using the R package “TCGAbiolinks” (v.2.28.3) (22). lncRNA and mRNA data were annotated using official website files followed by log2 (FPKM+1) calculations to make them more comparable. The somatic mutation analysis was all performed by R package “maftools” (v.2.16.0) (23). For DNA methylation data, β-values were log-transformed. The external validation cohorts ICGC-LIRI was obtained from the International Cancer Genome Consortium (ICGC) database (https://dcc.icgc.org/) and GSE14520 (24), GSE144269 (25), GSE141200 (26) and GSE141198 (26) were obtained from the NCBI Gene Expression Omnibus (GEO) database (https://www.ncbi.nlm.nih.gov/geo/). For genes with duplicates, the average value was taken. The samples were identified and only the data from the tumor tissue was kept.

Expression matrix and treatment response information for 67 HCC patients treated with sorafenib were extracted from the GSE109211 (27) dataset to assess whether subtypes were sensitive to sorafenib. GSE104580 containing 147 HCC patients treated with TACE was recruited to assess subtype sensitivity to TACE. In addition, GSE215011 (28) and GSE202069 (29), including 10 and 24 HCC patients respectively, were included to evaluate the association between molecular subtypes and immunotherapy response.

To further explore the proteomic characteristics of subtypes, protein expression matrix and corresponding clinical information of 151 HCC patients were obtained from the Clinical Proteomic Tumor Analysis Consortium (CPTAC) cohort (20). This dataset was utilized to validate subtype-specific molecular features at the proteomic level and to assess their clinical relevance.

The single-cell RNA sequencing (scRNA-seq) data were downloaded from the NCBI Gene GSE151530 (28), GSE156625 (29), GSE189903 (30) and GSE202642 (31). Among each sample, cells with fewer than 1000 UMI counts and genes expressed in less than 300 cells were excluded. In addition, a total of 273 genes associated with mitochondria, heat shock proteins and ribosomes were excluded to avoid expression artifacts from undetected noise and dissociation. After the quality filtering, 249012 cells were selected for the following analysis.

### 2.2 Data integration and molecular subtype identification

A new classification of HCC was established based on multi-omics data of mRNA expression, LncRNA expression, DNA methylation and somatic mutation data. The factors most associated with OS were extracted based on Cox regression survival analysis, ensuring that these factors were all *P ≤ 0.001*. Finally, 1000 mRNA, 100 lncRNA and 100 DNA methylation sites were recruited. Meanwhile, 11 genes with mutation frequencies greater than 3% were enrolled for multi-omics analysis.

To minimize noise while retaining important features, CPI and Gaps-statistics were used to obtain the optimal number of clusters. Subsequently, 10 algorithms (iClusterBayes, moCluster, CIMLR, IntNMF, ConsensusClustering, COCA, NEMO, PINSPlus, SNF and LRA) built into the R package “MOVICS” (32) are used to cluster the samples and the clustering results of different algorithms are integrated to improve the robustness of the clustering. In addition, the nearest template prediction (NTP) was run in external validation cohorts to verify the stability of the subtypes.

### 2.3 Survival analysis and comparison of clinical features

Survival analysis was conducted for different cohorts using the subtypes and Kaplan-Meier curves were plotted and Log-Rank tests were performed. At the same time, the differences in clinical characteristics were compared. In addition, in order to clarify the potential impact of subtypes on the prognosis of HCC patients, univariate and multivariate Cox survival analysis were conducted sequentially for different cohorts. The results were presented in forest plots and *P* < 0.05 considered significant.

### 2.4 Genomic characterization and tumor microenvironment analysis

The R package “maftools” (23) (v.2.16.0) was used to somatic mutation analysis. The ‘mafCompare’ function was used to identify differentially mutated genes between CS1 and CS2 and the top 20 were visualized by the ‘coOncoplot’ function. The ‘trinucleotideMatrix’ and ‘extractSignatures’ functions were used to identify retained characteristic mutation patterns in the cancer progression processes, thus enabling the interpretation of mutations as potential mutagenic processes. Mutant-allele tumor heterogeneity (MATH) and tumor mutation burden (TMB) was calculated for the TCGA-LIHC and ICGC-IRLI cohorts, and the mutation frequencies of TP53 and CNTTB1 were also compared between subtypes.

Download the snp6.na35.remap.hg38.subset.txt.gz file from GitHub (https://github.com/NCI-GDC/dnacopy-tool/) as a marker file. Split the masked copy number segment according to the subtype and use them as the segment file for CS1 and CS2 respectively. The maker file and segment file were uploaded to the GenePattern (https://www.genepattern.org/) website, while Human_hg38.UCSC.add_mir.160920.mat was selected as the reference file. Finally, the GISTIC 2.0 (33) module was run to investigate the CNVs of CS1 and CS2. After the run results are obtained, the R package “BSgenome.Hsapiens.UCSC.hg38” (v.1.4.5) was used to identify the chromosomal location of any amplification or deletion events.

In order to provide further clarification regarding the potential impact of CNV events on gene expression, genes corresponding to specific copy number variation events in CS1 and CS2 were extracted and integrated into an expression matrix, respectively. The RNA-seq (count format) data were analyzed using DEseq2 (34) (v.1.40.2), whereby genes with *P* < 0.05 and ∣log2FC∣≥ 1 was defined as differentially expressed genes (DEGs). Furthermore, the copy number values corresponding to the aforementioned differentially expressed genes were extracted from the file entitled “broad_data_by_genes.txt” and normalized to “Nor_CNV”. The student t-test was then used to identify “Nor_CNV” that differed significantly among subtypes and their corresponding genes were integrated with the DEGs in order to obtain the genes most likely to have altered expression due to copy number variation. Finally, the expression of genes was compared between subtypes and correlation curves were plotted between gene expression and “Nor_CNV”.

The tumor microenvironment (TME) of three independent study cohorts was decoded using xCell (35), quantiseq (36), TIMER (37) and MCPcounter (38) algorithms. The differences in cell type-specific immune infiltration scores between subtypes were analyzed using the limma (39) (v.3.56.2) algorithm. The results were then normalized and presented as heatmap.

### 2.5 Dimension reduction, integration and unsupervised clustering of single-cell RNA sequencing data

Single-cell RNA sequencing data from this study were analyzed uniformly using the R package “SCP” (v.0.5.6) (https://github.com/zhanghao-njmu/SCP). NormalizeData and ScaleData were used to normalize and scale the preprocessed data, respectively, while FindVariableGenes was used to identify highly variable genes. The “RunPCA” function was used to estimate the principal components (PCs). Then, the dimension range was set to 1:40, and the “RunUMAP” functions were used to perform the uniform manifold approximation and projection downscaling (UMAP). In order to eliminate the batch effect caused by the difference of sample sources, we used the “Harmony” function of the R package “harmony” (0.1.1) (40) for data integration. Set integration_method = “Harmony”, linear_reduction_dims_use = 1:50, and use the function FindNeighbors to allocate cells. In addition, set different resolutions and run FindClusters for unsupervised clustering. In conclusion, we displayed the clustering of cells at various resolutions in a tree format. We then selected the stable outcomes (cluster_resolution = 0.6) for further analysis. Based on published classical cell markers, six cell types were identified: B cells (*CD79A, CD79B*), Endothelial (*VWF, PECAM1*), Fibroblasts (*COL1A1, COL1A2*), Hepatocytes (*ALB, APOA2*), Myeloid (*LYZ, C1QB, S100A9*) and T/NK Cells (*CD1C, CD3D, CD3E*).

### 2.6 Identifying subtype-related subpopulations by integrating bulk and single-cell RNA sequencing data

We identified subtype-related subpopulations by the Scissor (41) algorithm. Briefly, we used CS1 and CS2 as the phenotype while collating a single-cell RNA sequencing data (scRNA-seq) expression matrix and bulk profiling data. The above three files were used as input data for Scissor, where CS2 was defined as a positive outcome and CS1 as a negative outcome. A regression model was built against the dichotomous variables to calculate the regression coefficients for each cell against the phenotype. Cells with negative regression coefficients are highly correlated with CS1, described as “Scissor_CS1”, cells with positive regression coefficients are highly correlated with CS2, described as “ Scissor_CS2”, and cells with zero regression coefficients are background cells, described as “NULL”.

### 2.7 Prediction of precise therapy strategies

Drug sensitivity analysis was performed using the oncoPredict (42) algorithm for subtypes, extracting results that were consistent across three independent cohorts for normalization, and ggplot2 for visualization. To assess the sensitivity of immunotherapy, the R package “easier” was used to calculate the Estimate Systems Immunotherapy response (EaSIeR) score (43). Based on the outcomes, the patients were classified into two groups, namely non-response (NR) and response (R). Bar graphs were used to compare the proportion of patients responding to treatment in different subtypes. At the same time, the Tumor Immune Dysfunction and Exclusion (TIDE) score were calculated using the TIDE algorithm under a Linux system (44). Additionally, four independent cohorts (GSE109211, GSE104580, GSE215011 and GSE202069) containing treatment information, were further used to compare differences in sensitivity between sorafenib, TACE and immunotherapy treatment between subtypes. For all comparisons, *P* < 0.05 was considered significant.

### 2.8 Construction and evaluation of the artificial intelligence-derived risk score

The AIDRS was developed following a well-established analytical framework from the R package “Mime” (v.0.0.0.9) (38), which integrates ten classical machine learning algorithms: random forest (RSF), elastic network (Enet), stepwise Cox (StepCox), CoxBoost, partial least squares regression for Cox (plsRcox), supervised principal components (superpc), generalized boosted regression models (GBM), survival support vector machine (survivalsvm), Ridge, and least absolute shrinkage and selection operator (Lasso). Among these, RSF, Lasso, CoxBoost, and different variants of StepCox (both directions and backward selection) were employed in the initial feature selection stately generating 117 distinct algorithmic combinations for model construction. AIDRS was developed using a structured multi-step process (1): Differential gene expression analysis was performed on both the training and validation cohorts, and input matrices were constructed by extracting genes that were differentially expressed in the three cohorts at the same time (2). Univariate Cox regression analysis was conducted using the coxph function from the R package “survival” (v.3.8-3) on both the training and validation cohorts. Candidate prognostic genes (CPGs) were identified based on *P ≤ 0.01* and consistent hazard ratios (HR > 1 or HR < 1) across both datasets (3). Feature selection and model fitting were performed using the 117 algorithmic combinations, where selected CPGs were incorporated into prognostic models trained on the Z-score normalized gene expression values (4). Model evaluation was conducted by computing risk scores for patients in the training, validation and independent test sets, utilizing the predict function from the respective model packages (5). Performance assessment was based on Harrell’s concordance index (C-index), which was calculated via univariate Cox regression analysis on the risk scores across all datasets (6). The final optimal model was automatically selected based on the highest average C-index across all three cohorts. The corresponding risk score derived from this model was defined as the AIDRS.

After construction, several strategies were used to further assess the predictive efficacy of the AIDRS in both the training and validation cohorts (1): Calculate the median risk score, categorize the HCC patients into high-risk and low-risk groups, plot the Kaplan-Meier curves and run the Log-Rank test to compare the differences in survival (2). Time-dependent ROC curve analysis was performed and the area under the curve (AUC) was calculated (3). Meta-analysis was performed for univariate Cox regression.

### 2.9 Multidimensional validation of AIDRS

The predictive efficacy of AIDRS was extensively and comprehensively validated using multi-omics data. Specifically, the AIDRS between subtypes in the training and validation cohorts were first compared to clarify differences between groups. Second, patients were grouped according to the median AIDRS and the Kaplan-Meier curves were plotted to compare survival differences. Subsequently, patients were differentiated on the basis of clinical characteristics and differences in AIDRS between groups were compared. In addition, patients in the TCGA-LIHC and ICGC-LIRC cohort were grouped according to whether TP53 and CTNNB1 were mutated or not, to verify the association between AIDRS and gene mutations. Further, the correlation between different treatment scores and AIDRS was calculated and correlation curves were plotted. Finally, AIDRS was calculated for scRNA-seq data using three methods (“Seurat”, “AUCell” and “UCell”), while comparing the intensity in different cell types.

### 2.10 Identification of key genes in AIDRS

The following steps were taken in order to identify overlapping genes included in the AIDRS model in the training and validation cohorts, along with multiple strategies to further identify key genes for AIDRS (1): AIDRS-associated genes were extracted from different study cohorts and overlapping genes were identified using veen plots (2). The multiplicity of differences in gene expression between subtypes will be compared (3). The prognostic hazard ratios of genes will be calculated based on univariate Cox regression (4). The Pearson correlation coefficient between gene expression and AIDRS will be calculated (5). Calculate the area under the curve (AUC) values of genes and compare the efficacy of genes in classifying subtypes (6). Group patients based on median gene expression, plot Kaplan-Meier curves and compare survival differences between groups using the Log-Rank test (7). Further validate the key genes based on scRNA-seq data.

### 2.11 Cell culture

The human HCC cell lines Bel-7402 and Hep-3B were obtained from the Cell Bank of Type Culture Collection of the Chinese Academy of Sciences (Shanghai, China). Both cell lines were maintained in Dulbecco’s Modified Eagle’s Medium (DMEM) (BasalMedia, Shanghai, China) supplemented with 10% fetal bovine serum (FBS, ExCell, Suzhou, China). Cells were incubated under standard culture conditions at 37°C with 5% CO_2_ in a humidified incubator.

### 2.12 Cell transfection and CEP55 knockdown

To achieve effective CEP55 knockdown, small interfering RNA (siRNA) specifically targeting CEP55 was designed and synthesized by RiboBio (Guangzhou, China). A non-targeting siRNA was used as the negative control (NC). Transfection efficiency was confirmed through quantitative real-time polymerase chain reaction (qRT-PCR).

### 2.13 Cell Counting Kit-8 assay

Transfected Bel-7402 and Hep-3B cells were seeded into 96-well plates and incubated under optimal conditions for 24, 48 and 72 h. The Cell Counting Kit-8 (CCK-8) (US Everbright, Suzhou, China) assay was performed according to the manufacturer’s protocol. Absorbance was measured at 450 nm using a microplate reader (Infinite F50, Tecan, Switzerland) to assess cell viability.

### 2.14 Colony formation assay

Transfected Bel-7402 and Hep-3B cells were trypsinized, counted and plated in 6-well plates at a density of 200 cells per well. The cells were cultured for 14 days to allow colony formation. Colonies were then fixed with 4% paraformaldehyde for 30 minutes, washed with phosphate-buffered saline (PBS), and stained with 0.1% crystal violet solution (Solarbio, Beijing, China) for 30 minutes. The number of colonies was counted and analyzed statistically.

### 2.15 Transwell migration assays

Cell migration ability was evaluated using transwell chambers with 24-μm pores. Briefly, 2.5 × 10^4^ transfected cells resuspended in serum-free DMEM were seeded into the upper chamber, while the lower chamber contained DMEM supplemented with 10% FBS. After 48 h of incubation at 37°C, non-migrated cells were carefully removed, and migrated cells were fixed, stained and counted under a light microscope.

### 2.16 Wound-healing assays

To further assess the migration capability of CEP55-silenced cells, a scratch wound healing assay was performed. Transfected cells were seeded into 6-well plates and grown to near confluence. A 200-μL pipette tip was used to create a straight scratch in the cell monolayer. Images were captured at 0 and 48 h to evaluate the wound closure rate, which was used to quantify the migratory potential of the cells.

### 2.17 Xenograft tumor model in nude mice

BALB/c nude mice (4–6 weeks old, male) were purchased from the Comparative Medicine Center of Yangzhou University (SYXK (Su) 2023-0019) and housed in a specific pathogen-free (SPF) facility with controlled temperature, humidity, and a 12 h light/dark cycle. All animal procedures were approved by the Dalian Medical University Animal Care and Ethics Committee (XL250423013) and were performed in accordance with the guidelines for the Care and Use of Laboratory Animals.

CEP55 knockdown and control groups were established against Bel-7402 and Hep-3B cell lines. Each group was injected subcutaneously into the dorsal axilla of nude mice (5 × 10^6^ cells in 100 µL PBS per mouse). Tumor growth and the health condition of the mice were monitored weekly. After 5 weeks, mice were euthanized by cervical dislocation, and tumors were excised, weighed, and measured. Tumor volume was calculated using the formula: V (cm^3^) = 1/2 × length × width^2^. The harvested tumor tissues were separated into two sections (1): preserved at -80°C for cryopreservation (2). fixed in a 4% paraformaldehyde solution.

### 2.18 Western blotting

Total protein was extracted from xenograft tumor tissues using a lysis buffer containing protease inhibitors. Protein concentration was determined using a BCA Protein Assay Kit (P0010, Beyotime Biotechnology, Shanghai, China). Equal amounts of protein were separated by 10% SDS-PAGE (S8010, Solarbio, Beijing, China) and transferred to PVDF membranes (ISEQ00010, Millipore, USA). The membranes were blocked with 5% non-fat milk and then incubated overnight at 4 °C with primary antibodies against CEP55 (1:1000, PA5-96976, Thermo, MA, USA) and GAPDH (1:500, ab8245, Abcam, Shanghai, China). After washing, the membranes were incubated with HRP-conjugated secondary antibodies at room temperature for 2 h. Protein bands were visualized using enhanced chemiluminescence (ECL) reagents (180-5001, Tanon, Shanghai, China). Band intensities were quantified using ImageJ software, and the relative expression levels of target proteins were normalized to GAPDH.

### 2.19 Immunohistochemistry

Tumor tissues were fixed in 4% paraformaldehyde, embedded in paraffin, and sectioned at 4 μm thickness. Sections were deparaffinized with xylene and rehydrated through graded ethanol. Antigen retrieval was performed using heated citrate buffer (C1010, Solarbio, Beijing, China) for 15 minutes. Endogenous peroxidase activity was blocked with 3% hydrogen peroxide for 20 minutes at room temperature. After blocking with normal serum, sections were incubated overnight at 4 °C with a primary antibody against CEP55 (1:50, 23891-1-AP, Proteintech, Wuhan, China) and Ki-67 (1:500, ab15580, Abcam, Shanghai, China), followed by incubation with an appropriate HRP-conjugated secondary antibody for 1 h at room temperature. DAB (Diaminobenzidine) was used for chromogenic detection, and hematoxylin was used for nuclear counterstaining. After dehydration and mounting, the stained sections were imaged using a brightfield microscope (NIB900, Leica Microsystems, Germany). The histochemistry score were evaluated semi-quantitatively.

### 2.20 Statistics and visualization

All statistical analyses for the figures were conducted using rstatix (v.0.7.2) and visualizations were generated with ggplot2 (v.3.4.3), except for methods where default tools were applied. Group comparisons were performed using parametric tests, such as Student’s t-test or Welch’s ANOVA test, provided the data followed normality and homogeneity of variance assumptions. In cases where data deviated from normality, non-parametric tests, including the Wilcoxon test or Kruskal-Wallis test, were employed, followed by Tukey’s *post-hoc* analysis. The Chi-square test was used to determine whether the sample distribution of a categorical variable is consistent. When identical statistical methods or color schemes are used in multiple parts of the manuscript, only the initial reference will include detailed annotations. All subsequent references will follow the same format and statistical approach as stated initially.

## 3 Results

### 3.1 Identify two molecular subtypes of HCC patients based on consensus clustering

**Figure 1:**
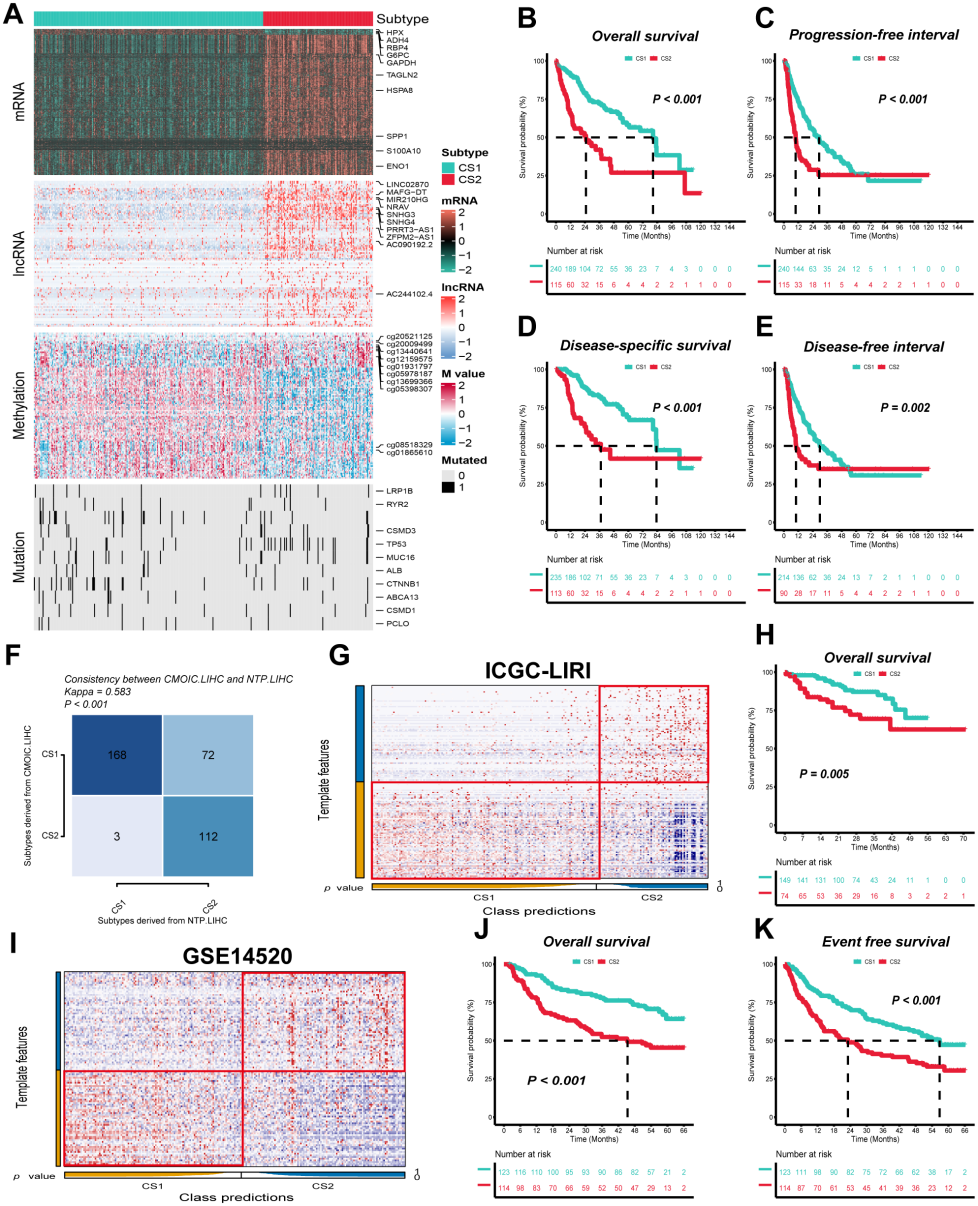
Two distinct molecular subtypes were identified through consensus clustering of multi-omics data, and clinical outcomes and stability were assessed. **(A)** Multi-omics features corresponding to CS1 and CS2 in the TCGA-LIHC cohort. M value, methylation value; CS, clustering subtype. **(B–E)** Kaplan–Meier curves corresponding to subtypes in the TCGA-LIHC cohort for overall survival, progression-free interval, disease-specific survival, and disease-free interval. **(F)** Consistency of subtype with nearest template prediction in the TCGA-LIHC cohort. **(G)** Evaluation of CS1 and CS2 subtypes in the ICGC-LIRI cohort. **(H)** Kaplan–Meier curves corresponding to subtypes in the GSE14520 cohort for overall survival. **(I)** Evaluation of CS1 and CS2 subtypes in the GSE14520 cohort. **(J, K)** Kaplan–Meier curves corresponding to subtypes in the GSE14520 cohort for overall survival and disease-free survival. Log-rank test was used in **(B, C, D, E, H, J, K)**.

After applying stringent data filtering, a total of 355 HCC patients with complete datasets across mRNA, lncRNA expression, DNA methylation, gene mutations and OS outcomes from the TCGA-LIHC cohort were selected for consensus clustering to identify molecular subtypes. Based on the optimal number of multi-omics clusters determined by the clustering prediction index (CPI) and Gap statistics, we identified two molecular subtypes for further analysis (**Supplementary Figure 1A**). Ten classical clustering algorithms available in the R package “MOVICs” (34) were employed to assign patients to these predefined molecular subtypes, followed by an ensemble consensus to ensure the robustness of the classification. The silhouette analysis further validated the clustering, demonstrating a moderate similarity among the samples in each subtype, with silhouette scores of 0.56 and 0.80 for CS1 and CS2, respectively (**Supplementary Figure 1B**). The distribution of the multi-omics data across these subtypes, along with associated clinicopathological features was shown in **Figure 1A**. For instance, CS1 displayed a higher DNA methylation profile, with patients carrying mutations in CTNNB1 primarily grouped in this subtype. CS2 was characterized by higher prevalence of mutations in TP53. In addition, most of the incorporated mRNA and lncRNA were highly expressed in CS2, including SPP1, S100A10, SNHG3, SNHG4 and so on.

The clinical prognostic outcome of HCC patients is a crucial factor in determining subsequent treatment options. We indicated that CS1 exhibited significantly superior overall survival (OS), progression-free interval (PFI), disease-specific survival (DSS) and disease-free interval (DFI) when compared to CS2, indicating a higher prognosis (*P ≤ 0.01*) (**Figures 1B–E**). In addition, the TCGA-LIHC cohort demonstrated that the nearest template prediction algorithm predictions were consistent with the original typing, thereby indicating stable and reliable CS subtypes and confirming the rationality of subtype extrapolation using nearest template prediction algorithm (**Figure 1F**).

### 3.2 Molecular subtypes further confirmed in independent cohort

In order to validate the external stability of the CS subtypes, the nearest template prediction algorithm was employed for the identification of subtypes against the ICGC-LIRI and GSE14520 cohorts. The ICGC-LIRI cohort comprised 223 HCC patients, of whom 149 were classified as CS1 and 74 as CS2, while the GSE14520 cohort contained 237 HCC patients, with 123 designated as CS1 and 114 as CS2 (**Figures 1G, I**). Furthermore, in both the ICGC-LIRI and GSE14520 cohorts, CS1, in comparison with CS2, exhibited superior overall survival (OS) and disease-free survival (PFI) (*P ≤ 0.01*) (**Figures 1H, J, K**).

### 3.3 Patients with different molecular subtypes face different clinicopathologic and functional features

**Figure 2:**
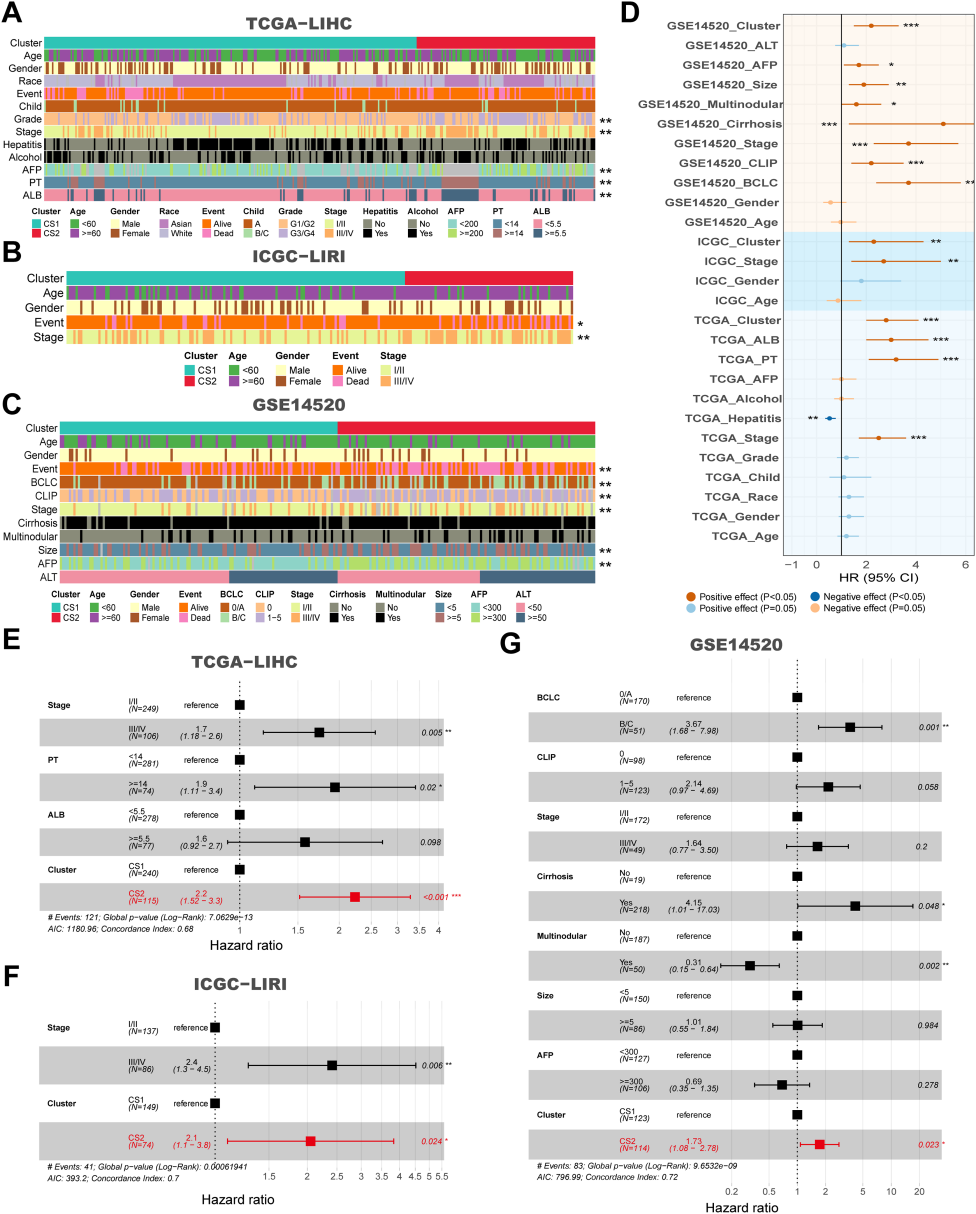
Clinical and molecular characteristics associated with subtypes across multiple cohorts, and their impact on survival. **(A)** Clinical features corresponding to CS1 and CS2 in the TCGA-LIHC cohort. **(B)** Clinical features corresponding to CS1 and CS2 in the ICGC-LIRI cohort. **(C)** Clinical features corresponding to CS1 and CS2 in the GSE14520 cohort. **(D)** Forest plot for univariate Cox of clinical variables and subtypes in the TCGA-LIHC, ICGC-LIRI and GSE14520 cohorts. **(E)** Hazard ratios for clinical features and CS subtypes in relation to overall survival based on multivariate Cox analysis in the TCGA-LIHC cohort. **(F)** Hazard ratios for clinical features and subtypes in relation to overall survival based on multivariate Cox analysis in the ICGC-LIRI cohort. **(G)** Hazard ratios for clinical features and subtypes in relation to overall survival based on multivariate Cox analysis in the GSE14520 cohort. *P < 0.05, **P ≤ 0.01, ***P ≤ 0.001.

In comparisons targeting clinicopathological features between subtypes, we found that CS2 patients in three independent cohorts (TCGA-LIHC, ICGC-LIRI and GSE14520) were mostly in advanced tumor stage, along with higher alpha-fetoprotein (AFP), longer prothrombin time (PT), and larger tumor size compared to CS1 (*P < 0.05*) (**Figures 2A–C**). Second, in the univariate Cox regression of prognostic factors for HCC patients, CS subtypes were shown to be a prognostic risk factor in both the training and validation cohorts, with hazard ratios of 2.8, 2.3 and 2.2 in that order (*P < 0.05*) (**Figure 2D**). The hazard ratios of CS subtypes were similar to the tumor stage, which is often used to evaluate the patient’s prognosis in the clinic and were significantly superior to those of AFP, albumin (ALB) and PT (*P < 0.05*) (**Figure 2D**). In addition, after further incorporating the statistically significant prognostic factors into the multivariate Cox regression, we found that the statistical efficacy of a variety of metrics, including AFP, ALB and PT, was significantly reduced (*P < 0.05*) (**Figures 2E–G**). In contrast, CS subtypes and tumor stage were statistically different in the three independent cohorts, with hazard ratios for CS subtypes being: 2.2, 2.1 and 1.73, respectively (*P < 0.05*) (**Figures 2E–G**).

The differential expression analysis among subtypes yielded 4562, 2638 and 556 differentially expressed genes in the three study cohorts, respectively. Of these, 73 were concurrently expressed upregulated genes and 99 were expressed downregulated genes (**Supplementary Figure 1C**). GO enrichment analysis subsequently revealed that 172 DEGs were closely associated with metabolism-related biological processes, including xenobiotic metabolic process, small molecule metabolic process and oxoacid metabolic process (*P < 0.05*). Additionally, these genes were found to be actively involved in immune responses, such as positive regulation of immune system process (*P < 0.05*) (**Supplementary Figure 1D**). The KEGG analysis indicated that the DEGs were significantly enriched in pathways such as the Toll-like receptor signaling pathway, metabolic pathways and the IL-17 signaling pathway (*P < 0.05*) (**Supplementary Figure 1E**). Furthermore, an analysis of the differences in feature scores across three distinct study cohorts revealed that scores associated with immune activation-related pathways were significantly elevated in CS1, including “TMEscoreA_CIR”, “TIP_Recognition_of_cancer_cells_by_T_cells_1”, “TIP_Infiltration_of_immune_cells_into_tumors_2” and so on (*P < 0.05*) (**Supplementary Figure 1F**). Conversely, scores for biological metabolic pathways such as “Tyrosine_Metabolism”, “Tryptophan_Metabolism”, “Steroid_Hormone_Metabolism” were significantly lower in CS1 compared to CS2 (*P < 0.05*) (**Supplementary Figure 1G**). Overall, CS1 exhibited significant immune activity, while CS2 was closely associated with biological metabolic pathways.

### 3.4 Genomic alterations with different molecular subtypes

**Figure 3:**
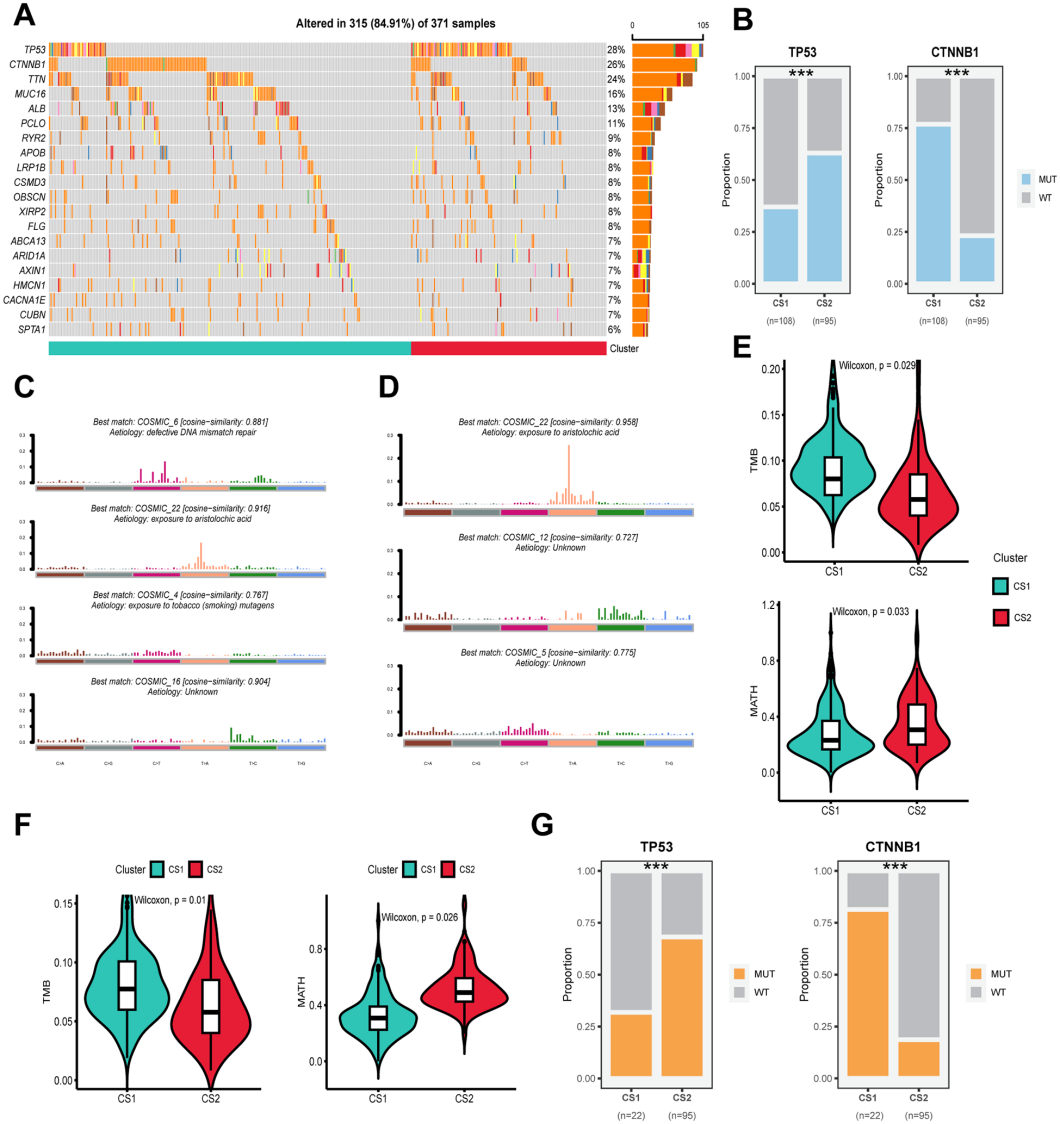
Genomic alterations, mutation signatures and mutational burden in subtypes across cohorts. **(A)** Oncoplot showing the distribution of somatic mutations across the most frequently altered genes for CS1 and CS2 in the TCGA-LIHC cohort. **(B)** Proportions of mutations in TP53 and CTNNB1 for CS1 and CS2 in the TCGA-LIHC cohort. **(C)** The best matching COSMIC mutational signatures (with similarity scores) for CS1. **(D)** The best matching COSMIC mutational signatures (with similarity scores) for CS2. **(E)** Violin plots showing the distribution of tumor mutational burden (TMB) in CS1 and CS2 subtypes in the TCGA-LIHC cohort. **(F)** Violin plots showing the distribution of TMB in CS1 and CS2 subtypes in the ICGC-LIRC cohort. **(G)** Mutation status of TP53 and CTNNB1 in CS1 and CS2 subtypes, showing the proportion of wild-type (WT) and mutant (MUT) alleles for each gene in the different clusters. Wilcoxon test was used in **(E, F)** Chi-square test was used in **(B, G)** ***P ≤ 0.001.

Following the sorting of the top twenty genes according to mutation frequency, it was established that the top three mutated genes in the TCGA-LIHC cohort were TP53 (28%), CTNNB1 (26%) and TTN (24%) (**Figure 3A**). Twenty genes were identified as mutated in both CS1 and CS2, and no specific genes were found to be mutated only in one subtype. However, a significant variation in the frequency of gene mutation was observed among the different subtypes. For instance, the mutation frequency of the CTNNB1 gene was approximately 75% in CS1, which is considerably higher than the 25% observed in CS2. Conversely, mutations in the TP53 gene were present in about 67.5% of all CS2 individuals, compared to only about 47.5% of individuals with mutations in CS1 (*P ≤ 0.001*) (**Figure 3B**). Furthermore, CS2 exhibited higher TMB and lower MATH compared to CS1 (*P < 0.05*) (**Figure 3E**). Significant differences in TP53 and CTNNB1 mutation frequency, TMB and MATH between subtypes were likewise confirmed in the validation cohort ICGC-LIRI (*P < 0.05*) (**Figures 3F, G**). In the analysis of mutations against genes, we found that mutations in CS1 were enriched for defective DNA mismatch repair features (COSMIC_6), exposure to aristolochic acid (COSMIC_22) and exposure to tobacco (smoking) mutagens (COSMIC_4), whereas mutations in CS2 were mainly enriched for exposure to aristolochic acid (COSMIC_22) (**Figures 3C, D**).

**Figure 4:**
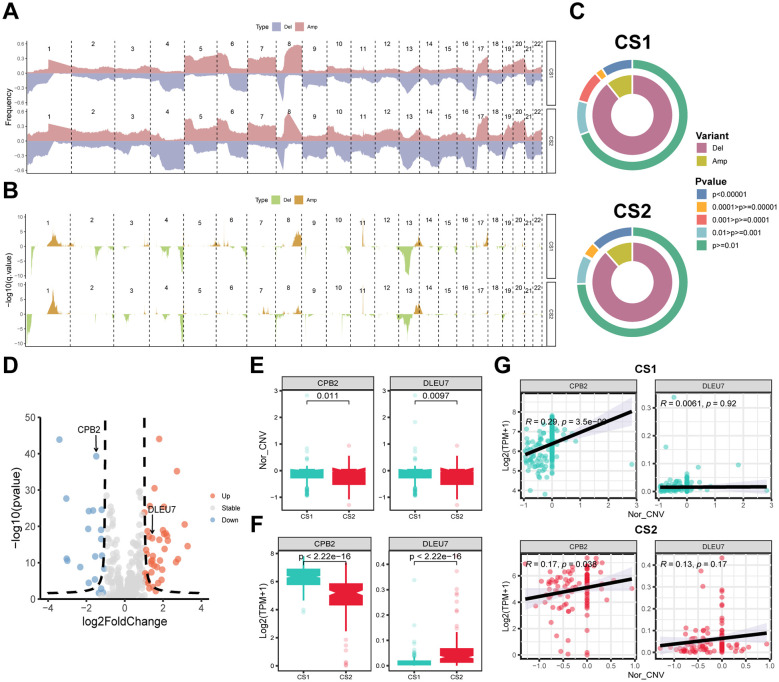
Copy number alterations (CNA), gene expression and their correlation with subtypes. **(A)** Frequency plot showing the distribution of CNA across chromosomes for CS1 and CS2, with deletions (Del) and amplifications (Amp) indicated. **(B)** Statistical significance of CNA events with -log10 p-values shown for each region. **(C)** Circular plots depicting the distribution of CNA variants (Del and Amp) and their statistical significance across CS1 and CS2. Colors represent different levels of significance. **(D)** Volcano plot showing differentially expressed genes (DEGs). Red indicates upregulated genes and blue indicates downregulated ones. **(E)** Box plots showing the copy number variation (CNV) score of CPB2 and DLEU7 in CS1 and CS2. **(F)** Box plots presenting the gene expression of CPB2 and DLEU7 in CS1 and CS2. **(G)** Correlation analysis between CPB2 and DLEU7 gene expression and CNV score in CS1 and CS2. Pearson correlation coefficients and p-values are indicated. Wilcoxon test was used in **(E, F)**.

For CNVs, the frequency of mutation events on different chromosomes and the corresponding p-values were calculated separately after grouping them according to subtypes. The results showed a higher frequency of gene copy number deletions on chromosomes 4 and 13–16 and a lower frequency of gene copy number duplications on chromosomes 5 and 8 in CS2 compared to CS1 (**Figures 4A, B**). Concurrently, the statistical efficacy of copy number variation events in CS1 and CS2 was inadequate, particularly in the context of gene copy number deletion events (**Figure 4C**). In the subsequent integrated analysis, it was found that CNV events involved a total of 415 genes, of which 62 genes were differentially expressed between subtypes, containing 42 upregulated and 20 downregulated genes (**Figure 4D**). Among them, only CPB2 and DLEU7 genes showed simultaneous differences in expression and copy number values between subtypes (*P* < 0.05) (**Figures 4E–G**). Furthermore, the correlation analysis for gene expression and copy number values revealed a consistent positive correlation for the CPB2 gene in both CS1 and CS2 (*P* < 0.05), while the correlation for the DLEU7 gene did not satisfy the statistical difference (*P* ≥ 0.05) (**Figure 4G**).

### 3.5 CS1 has abundant immune infiltration and CS2 has dense tumor cells

**Figure 5:**
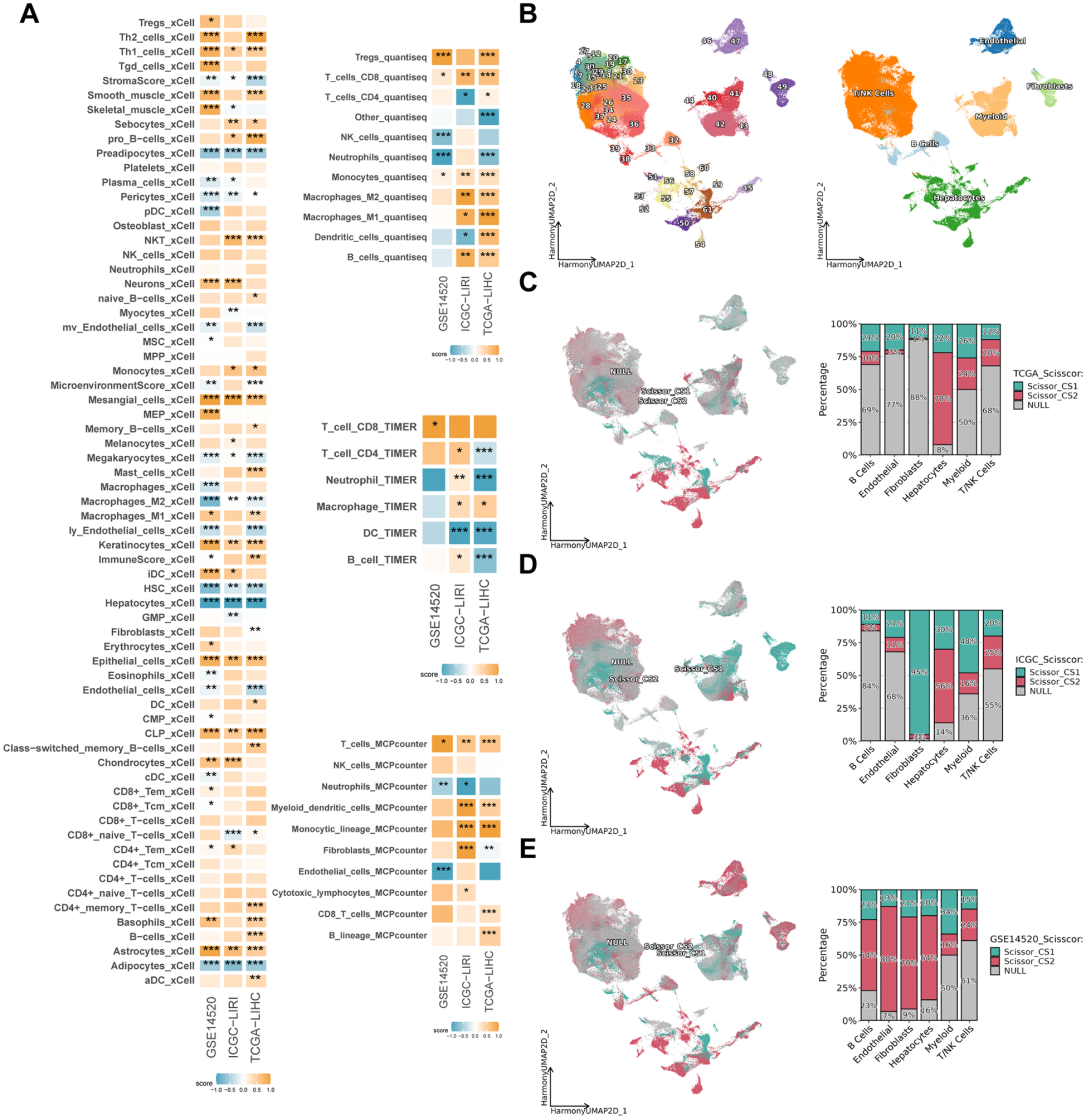
Immune infiltration and subtypes distribution across different datasets. **(A)** Heatmap showing the enrichment scores of various immune and stromal cell types in CS1 and CS2 across TCGA-LIHC, ICGC-LIRI and GSE14520 cohorts. The color intensity represents the degree of enrichment. **(B)** Uniform manifold approximation and projection (UMAP) of scRNA-seq data, depicting different cell populations across the dataset, with major cell types labeled. **(C–E)** Scissor-based subtypes distribution of CS1 and CS2 in TCGA-LIHC, ICGC-LIRI and GSE14520 cohorts. The left panels display UMAP projections with cells color-coded by subtype (CS1: red, CS2: cyan, NULL: gray). The right bar plots illustrate the proportion of different cell types within each subtype. *P < 0.05, **P ≤ 0.01, ***P ≤ 0.001.

The present study evaluated the cell types in the tumor microenvironment of HCC patients in three independent study cohorts, utilizing four distinct inverse convolution algorithms. The analysis revealed that CS1 exhibited a higher abundance of CD8^+^ T, CD4^+^ T, NK Cells and M1-type macrophages, indicative of a more pronounced immune cell infiltration compared to CS2 (*P < 0.05*). Conversely, CS2 demonstrated a higher prevalence of non-immune cells, including hepatocytes, endothelial cells, fibroblasts and pericytes, exhibiting significant disparities among the various subtypes (*P < 0.05*) (**Figure 5A**).

Following the initial quality control and dimensionality reduction clustering, a single-cell atlas of HCC patients containing 249012 cells with 39 cell subpopulations was established. Initially, the cell subpopulations were separated from each other according to the sample source, which exhibited a significant batch effect (**Supplementary Figure 2A**). Following data integration by the “harmony” algorithm (40), the distributions of cells from different samples overlapped with each other in the two-dimensional space, thereby effectively avoiding the generation of aberrant cell clusters from the sample source (**Supplementary Figure 2B**). Subsequently, the cells were distinguished into six categories based on classical marker genes (**Supplementary Figure 2C**; **Figure 5B**). Following the mapping of CS subtypes to single-cell atlases based on the “Scissor” algorithm (41), it was found that the results in the three study cohorts varied greatly (**Figures 5C–E**). For example, CS1 was found to be concentrated in the fibroblast subpopulation in the ICGC-LIRI cohort, but not in the TCGA-LIHC and GSE14520 cohorts. A similar observation was made in the GSE14520 cohort, where CS2 was found to be concentrated in B cells, endothelial cells, fibroblasts and hepatocyte subpopulations. However, its distribution was not found to be simultaneous in the other two cohorts. It is noteworthy that all three study cohorts exhibited a centralized distribution of CS2 in the hepatocyte subpopulation, with percentages of 70%, 56%, and 64%, respectively (**Figures 5C–E**). This was significantly higher than the percentage of CS1. This finding indicates that CS2 exhibits a strong association with hepatocyte subpopulations, the inverse convolution results that is further validated by this conclusion.

### 3.6 CS1 is sensitive to immunotherapy, CS2 is more suitable for sorafenib and TACE

**Figure 6:**
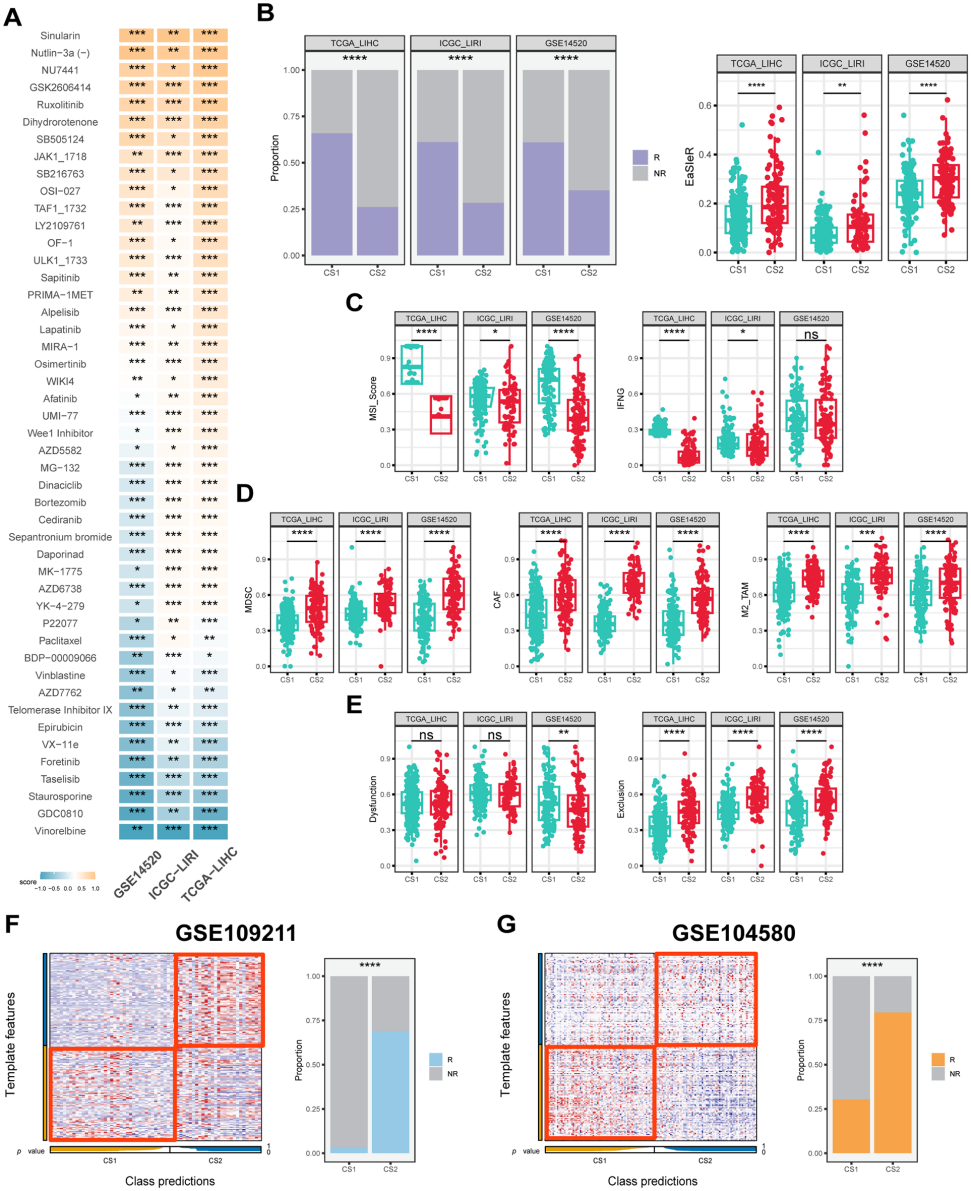
Drug response, immune score and predictive classification across different datasets. **(A)** Heatmap of drug response in CS1 and CS2 across the TCGA-LIHC, ICGC-LIRI and GSE14520 cohorts. The color represents drug response in each subtype, with yellow signifies CS1 sensitivity and blue signifies CS2 sensitivity. **(B)** Proportion of immunotherapy response and boxplots of EasleR score in CS1 and CS2 across the TCGA-LIHC, ICGC-LIRI and GSE14520 cohorts. **(C)** Boxplots showing MSI score and INFG in CS1 and CS2 across the TCGA-LIHC, ICGC-LIRI and GSE14520 cohorts. **(D)** Boxplots showing MDSC, CAF and M2-TAMs in CS1 and CS2 across the TCGA-LIHC, ICGC-LIRI and GSE14520 cohorts. **(E)** Boxplots showing T cell dysfunction and exclusion score in CS1 and CS2 across the TCGA-LIHC, ICGC-LIRI and GSE14520 cohorts. **(F)** Evaluation of CS1 and CS2 in the GSE109211 cohort. **(G)** Evaluation of CS1 and CS2 in the GSE104580 cohort. R indicates response, NR indicates no response. Wilcoxon test was used in **(A–E)** Chi-square test was used in **(B, F, G)** ns *P* ≥ 0.05, *P < 0.05, **P ≤ 0.01, ***P ≤ 0.001, ****P ≤ 0.0001.

In order to ascertain the most appropriate treatment for the various subtypes of HCC, the oncoPredict algorithm (42) was utilized to evaluate patients’ therapeutic sensitivity. The findings revealed that CS1 exhibited high sensitivity to treatment with drugs such as nutlin-3 and ruxolitinib (*P* < 0.05), while microtubule inhibitors such as paclitaxel and vinblastine appeared to be more suitable for the treatment of CS2 (**Figure 6A**). Furthermore, in the analysis for the sorafenib treatment cohort GSE109211, patients categorized as CS2 demonstrated a treatment response rate of approximately 70%, whereas CS1 was even less than 5% (**Figure 6F**). Similarly, in the TACE treatment cohort GSE104580, about 75% of CS2 belonged to the treatment-responsive population, and far fewer, just about 27% for CS1 (*P ≤ 0.0001*) (**Figure 6G**).

In terms of predicting immunotherapy response in HCC patients, CS2 had higher EaSleR score than CS1, with a significant difference between the two (*P* < 0.05) (**Figure 6B**). Accordingly, CS1 had a similar response rate of approximately 60% across the three study cohorts, while CS2 had a peak response rate of only 30% (*P ≤ 0.0001*) (**Figure 6B**). In addition, the TIDE algorithm (44) was used to further evaluate a number of predictors in HCC patients that have been shown to potentially influence immunotherapy response. The results showed that CS1 had higher MSI score and IFNG compared to CS2, which was consistent across the three study cohorts (*P* < 0.05) (**Figure 6C**). CS2 had higher levels of MDSC, CAF and M2-type TAM, which are associated with the immunosuppressive microenvironment (*P* < 0.05) (**Figure 6D**). Meanwhile, CS1 had a lower cytotoxic T-cell dysfunction and exclusion score, suggesting its potential immune-activating activity (*P* < 0.05) (**Figure 6E**). Notably, the results of the explorations for the two real-world immunotherapy cohorts were consistent with our computational predictions, confirming that CS1 patients responded significantly better to immunotherapy than CS2 patients. Specifically, the majority of CS1 patients in GSE215011 and GSE202069 demonstrated a positive response to treatment, with response rates as high as 75% and 80% for CS1 patients compared to less than 25% for CS2 patients, respectively (*P ≤ 0.0001*) (**Supplementary Figures S2D, E**).

### 3.7 Integrated machine learning algorithms to develop artificial intelligence-driven risk score

**Figure 7:**
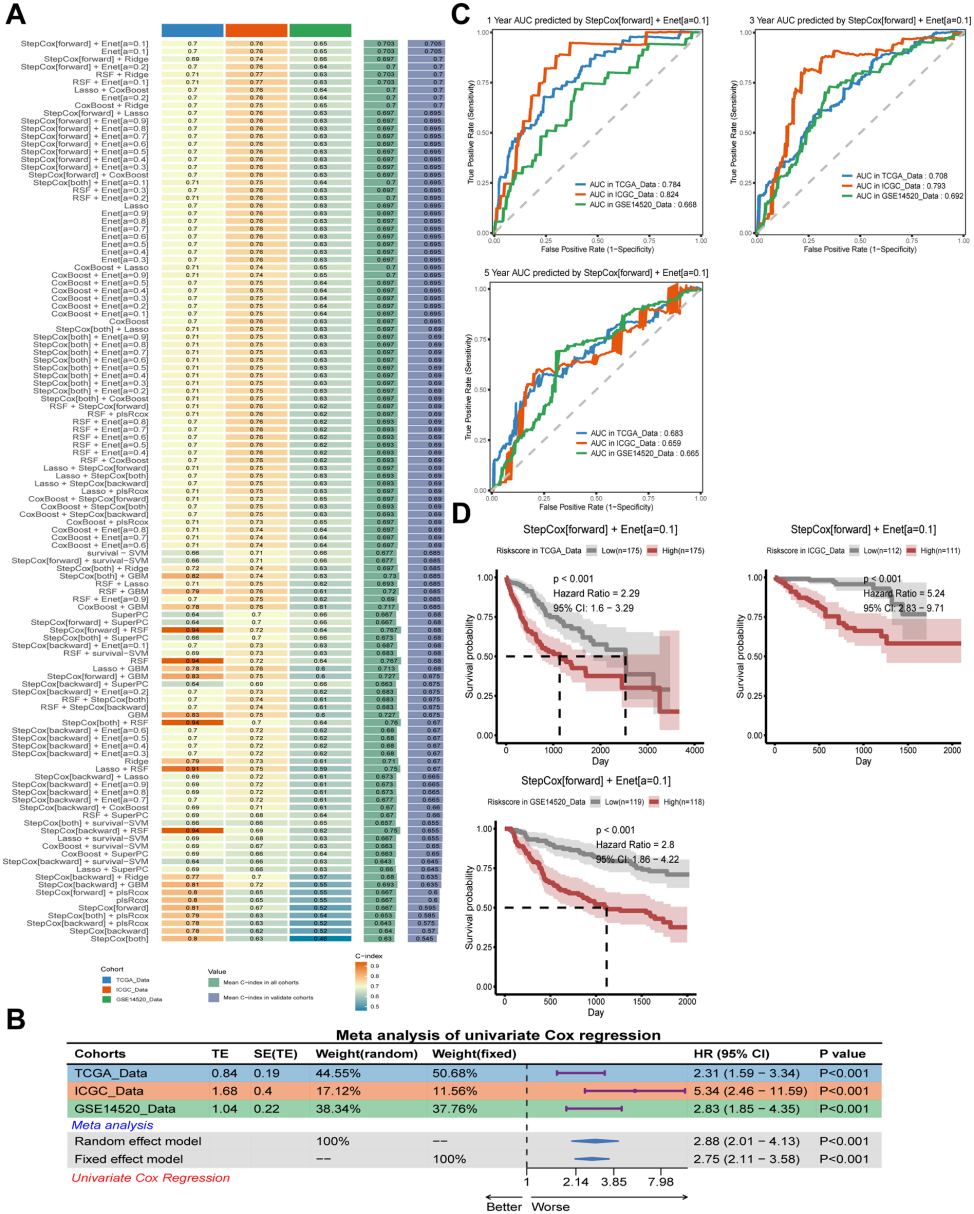
Construction and evaluation of prognostic models across different datasets. **(A)** C-index of each model among different datasets sorted by the average of C-index in validation cohorts. **(B)** Meta-analysis of univariate Cox result of the best model StepCox[forward]+Ent[a=0.1] across the TCGA-LIHC, ICGC-LIRC and GSE14520 cohorts. **(C)** Receiver operating characteristic (ROC) curves showing the prediction performance of the StepCox[forward]+Ent[a=0.1] model for 1-year (top left), 3-year (top right), and 5-year (bottom left) survival data across the TCGA-LIHC, ICGC-LIRC and GSE14520 cohorts. **(D)** Kaplan-Meier curves showing the survival probability for high-risk and low-risk groups predicted by the risk score calculated by StepCox[forward]+Ent[a=0.1] model across TCGA-LIHC (left), ICGC-LIRC (right), and GSE14520 (bottom) cohorts. Log-rank test was used in **(D)**.

A comprehensive analysis of these 172 overlapping DEGs was conducted using 10 machine learning algorithms, which resulted in the creation of 117 prognostic prediction models. The consistency indices of these models were then calculated for each cohort and their mean values were determined within the overall study cohort. Of all the models, the StepCox[forward]+Ent[a=0.1] model demonstrated the most consistent prognostic prediction efficacy, exhibiting the highest average consistency indices of 0.703 and 0.075 (**Figure 7A**).

In the univariate Cox regression analysis for the training and validation cohorts, the StepCox[forward]+Ent[a=0.1] model corresponded to hazard ratio (HR) of 2.31, 5.34, and 2.83, respectively (*P ≤ 0.001*) (**Figure 7B**). Meanwhile, in the Meta- analysis of HR for the StepCox[forward]+Ent[a=0.1] model across the three study cohorts, the random-effects model and the mixed-effects model corresponded to HR of 2.88 and 2.75, respectively, and met the statistical differences (*P ≤ 0.001*) (**Figure 7B**). In addition, the prognostic predictive efficacy of the StepCox[forward]+Ent[a=0.1] model was found to be superior and accurate at 1, 3, and 5 years, respectively, and relatively stable across cohorts. The corresponding AUC values ranged from 0.824 to 0.659 (**Figure 7C**). It is noteworthy that the average AUC of the model gradually decreased with the prolongation of follow-up time, which is consistent with the actual situation.

Following the determination of the optimal prognostic model, the prognostic risk score, herein referred to as AIDRS, was calculated for each patient with HCC. Subsequent analyses revealed that patients with HCC exhibiting a high AIDRS demonstrated a worse prognosis in both the training and validation cohorts, corresponding to prognostic HR of 2.29, 5.24, and 2.8, respectively (*P ≤ 0.001*) (**Figure 7D**). This finding suggests that AIDRS is a key risk factor for the prognosis of patients with HCC.

### 3.8 Association between AIDRS and patient prognosis, clinicopathological features, genomic alterations, and personalized therapy

**Figure 8:**
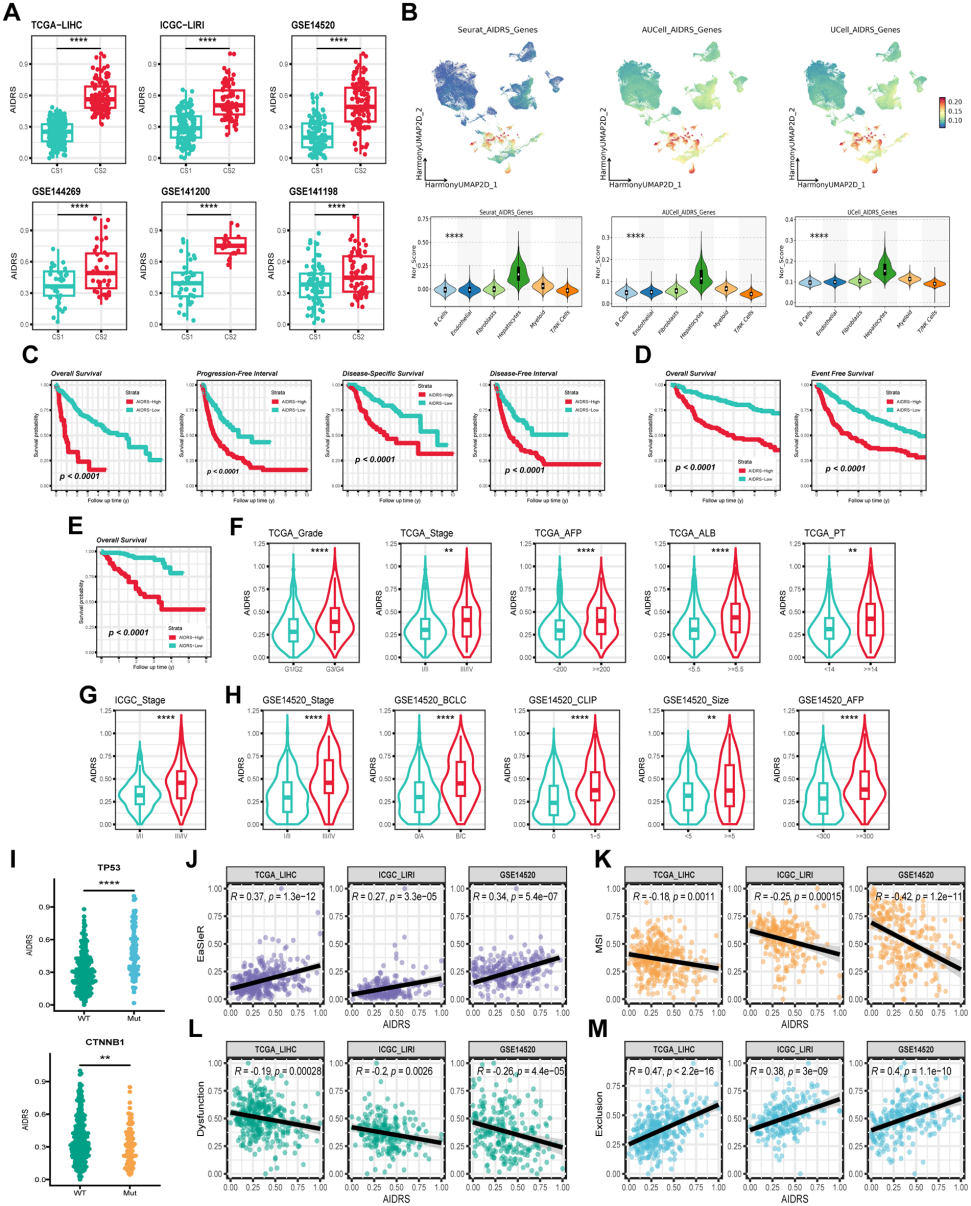
Correlation of AIDRS with clinicopathological features, immune score, and survival outcomes across different datasets. **(A)** Boxplots displaying the AIDRS across the CS1 and CS2 for the TCGA-LIHC, ICGC-LIRI and GSE14520 cohorts. **(B)** UMAP plots (top) and violin plots (bottom) showing the expression distribution of AIDRS-related genes across the TCGA-LIHC, ICGC-LIRI and GSE14520 cohorts. **(C)** Kaplan-Meier curves showing the overall survival, progression-free interval, disease-specific survival and disease-free interval for high- and low-risk groups in the TCGA-LIHC cohort. **(D)** Kaplan-Meier curves showing the overall survival and progression-free interval in the ICGC-LIRI cohort. **(E)** Kaplan-Meier curve showing the overall survival in the GSE14520 cohort. **(F)** Violin plots illustrating the distribution of AIDRS based on clinicopathological features such as Grade, Stage, AFP, ALB and PT across the TCGA-LIHC cohort. **(G)** Violin plots comparing AIDRS across Stage in the ICGC-LIRI cohort. **(H)** Violin plots showing AIDRS based on clinicopathological features such as Stage, CLIP, AFP and tumor size across the GSE14520 cohort. **(J)** Scatter plots showing the correlation between AIDRS and EasleR score in the TCGA-LIHC, ICGC-LIRI and GSE14520 cohorts. **(K)** Scatter plots showing the correlation between AIDRS and MSI score in the in the TCGA-LIHC, ICGC-LIRI and GSE14520 cohorts. **(L)** Scatter plots showing the correlation between AIDRS and T cell dysfunction score in the in the TCGA-LIHC, ICGC-LIRI and GSE14520 cohorts. **(M)** Scatter plots showing the correlation between AIDRS and T cell exclusion score in the in the TCGA-LIHC, ICGC-LIRI and GSE14520 cohorts. Log-rank test was used in **(C, D, E)** Wilcoxon test was used in **(A, F, G, H, I)** Welch’s ANOVA test was used in **(B)** **P ≤ 0.01, ****P ≤ 0.0001.

In each of the six mutually independent study cohorts, CS2 exhibited significantly higher AIDRS than CS1 (*P ≤ 0.0001*) (**Figure 8A**). Meanwhile, AIDRS was calculated separately for scRNA-seq data using three different algorithms: “Seurat”, “AUcell” and “Ucell”. The results demonstrated that hepatocytes exhibited higher AIDRS (*P ≤ 0.0001*) (**Figure 8B**). This finding was consistent across the TCGA-LIHC, ICGCI-LIRI and GSE14520 cohorts. Subsequently, survival analysis revealed that patients in the high AIDRS group corresponded to more fatal events and had a worse prognosis, and this finding was consistent across the TCGA-LIHC, ICGCI-LIRI and GSE14520 cohorts (*P ≤ 0.0001*) (**Figures 8C–E**).

Further analyses addressed AIDRS and clinicopathological features, revealing that patients with advanced tumors exhibited higher AIDRS (*P ≤ 0.01*) (**Figures 8F–H**). Similarly, prognostic risk factors such as AFP, PT and tumor volume were approximately significant, with higher AIDRS correlating with increased risk (*P ≤ 0.01*) (**Figures 8F–H**). Furthermore, patients with TP53 mutant HCC patients exhibited a higher AIDRS in comparison to those with the wild type (*P ≤ 0.0001*) (**Figure 8I**). Conversely, patients with CTNNB1 mutant patients demonstrated a lower AIDRS (*P ≤ 0.01*) (**Figure 8I**). The correlation analysis between AIDRS and immunotherapy response-related scores revealed that higher AIDRS was associated with higher EaSleR score and T-cell exclusion score, and lower MSI score and T-cell dysfunction score (*P ≤ 0.01*) (**Figures 7J–M**). These findings suggest that higher AIDRS is associated with a limited benefit from immunotherapy.

### 3.9 CEP55 has good predictive efficacy and positively influences patient prognosis

**Figure 9:**
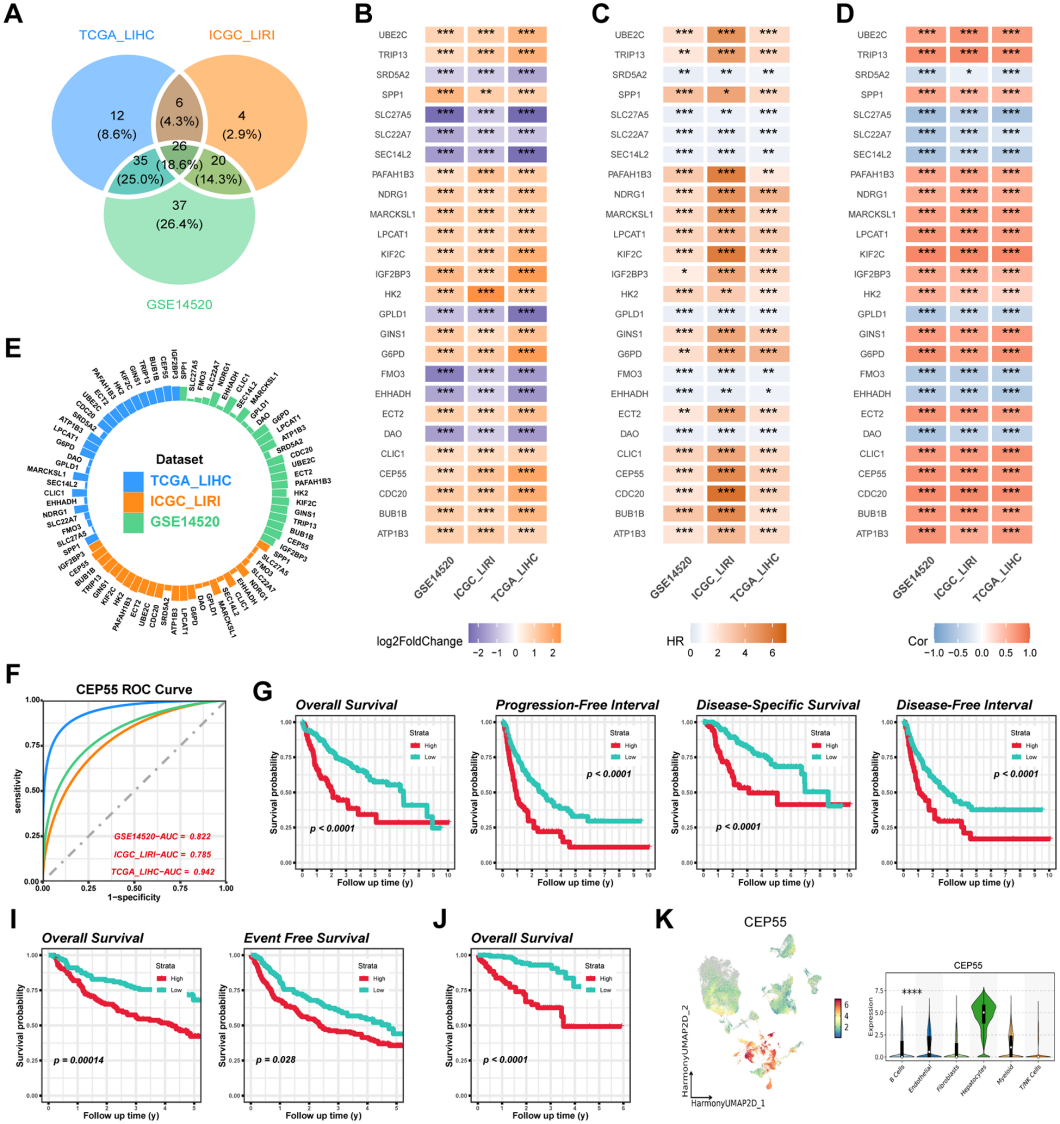
Identification and validation of CEP55 as a prognostic biomarker across multiple datasets. **(A)** Overlapping differentially expressed genes (DEGs) among the TCGA-LIHC, ICGC-LIRI and GSE14520 cohorts. **(B)** Log2 fold change (log2FC) values of overlapping DEGs across the TCGA-LIHC, ICGC-LIRI and GSE14520 datasets. **(C)** Univariate Cox regression for overlapping DEGs associated with survival outcomes in TCGA-LIHC, ICGC-LIRI and GSE14520 cohorts. **(D)** Correlation heatmap showing the association of AIDRS and overlapping DEGs across the datasets. The color intensity represents the strength of the correlation. **(E)** Circular plot displaying the AUC value of overlapping DEGs, with each dataset (TCGA-LIHC, ICGC-LIRI and GSE14520) represented in different colors. **(F)** ROC curves illustrating the predictive accuracy of CEP55 in TCGA-LIHC, ICGC-LIRI and GSE14520 datasets. **(G)** Kaplan-Meier curves for overall survival, progression-free interval, disease-specific survival and disease-free interval of high and low CEP55 expression groups in the TCGA-LIHC cohort. **(H)** Kaplan-Meier curves for overall survival and event-free survival of high and low CEP55 expression groups in the ICGC-LIRI dataset. **(I)** Kaplan-Meier curve for overall survival in the GSE14520 dataset, showing significant survival differences between high and low CEP55 expression groups. **(J)** UMAP plot displaying the expression of CEP55 across different cell populations. The violin plot on the right shows the distribution of CEP55 expression across major cell types. Log-rank test was used in **(G, I, J)** Welch’s ANOVA test was used in **(K)** *P < 0.05, **P ≤ 0.01, ***P ≤ 0.001, ****P ≤ 0.0001.

The genes corresponding to the StepCox[forward]+Ent[a=0.1] model in the three study cohorts were extracted, 79, 56, and 118, respectively, with 26 overlapping genes among the three (**Figure 9A**). Differential expression analysis of the subtypes showed that 18 genes in CS2, including CEP55, showed consistent upregulation of expression in the three study cohorts, while the remaining 8 genes were down-regulated (*P < 0.05*) (**Figure 9B**). Subsequent univariate Cox regression and correlation analysis revealed that all genes with upregulated expression in CS2 belonged to the prognostic risk factors for HCC patients and were significantly positively associated with AIDRS (*P < 0.05*) (**Figures 9C, D**). Conversely, genes downregulated in CS2 showed protective factors for HCC prognosis and were significantly negatively correlated with AIDRS (*P < 0.05*) (**Figures 9C, D**). Furthermore, the proteomics cohort CPTAC revealed that patients exhibiting low CEP55 expression exhibited significantly prolonged overall survival when compared to those exhibiting high CEP55 expression (*P ≤ 0.01*) (**Supplementary Figure 2F**). Concurrently, patients in the CEP55 high-expression exhibited elevated AIDRS (*P < 0.05*) (**Supplementary Figure 2G**), and CEP55 protein expression levels demonstrated a significant positive correlation with AIDRS (*R = 0.27, P* < *0.05*) (**Supplementary Figure 2H**).

In analyses targeting the predictive efficacy of subtypes, CEP55 exhibited higher AUC compared to other genes in both the training and validation cohorts, with 0.942, 0.785, and 0.822, respectively (**Figures 9E, F**). For subsequent survival analyses, we found that HCC patients with high expression of CEP55 were more likely to experience a fatal event during the follow-up period, which was consistent among the three cohorts of independent studies of each other (*P < 0.05*) (**Figures 9G–J**). Furthermore, scRNA-seq data indicated that CEP55 was expressed at the highest level in hepatocytes (*P ≤ 0.0001*) (**Figure 9K**).

### 3.10 Knockdown of CEP55 inhibited cell proliferation, migration and invasion of HCC cells

**Figure 10:**
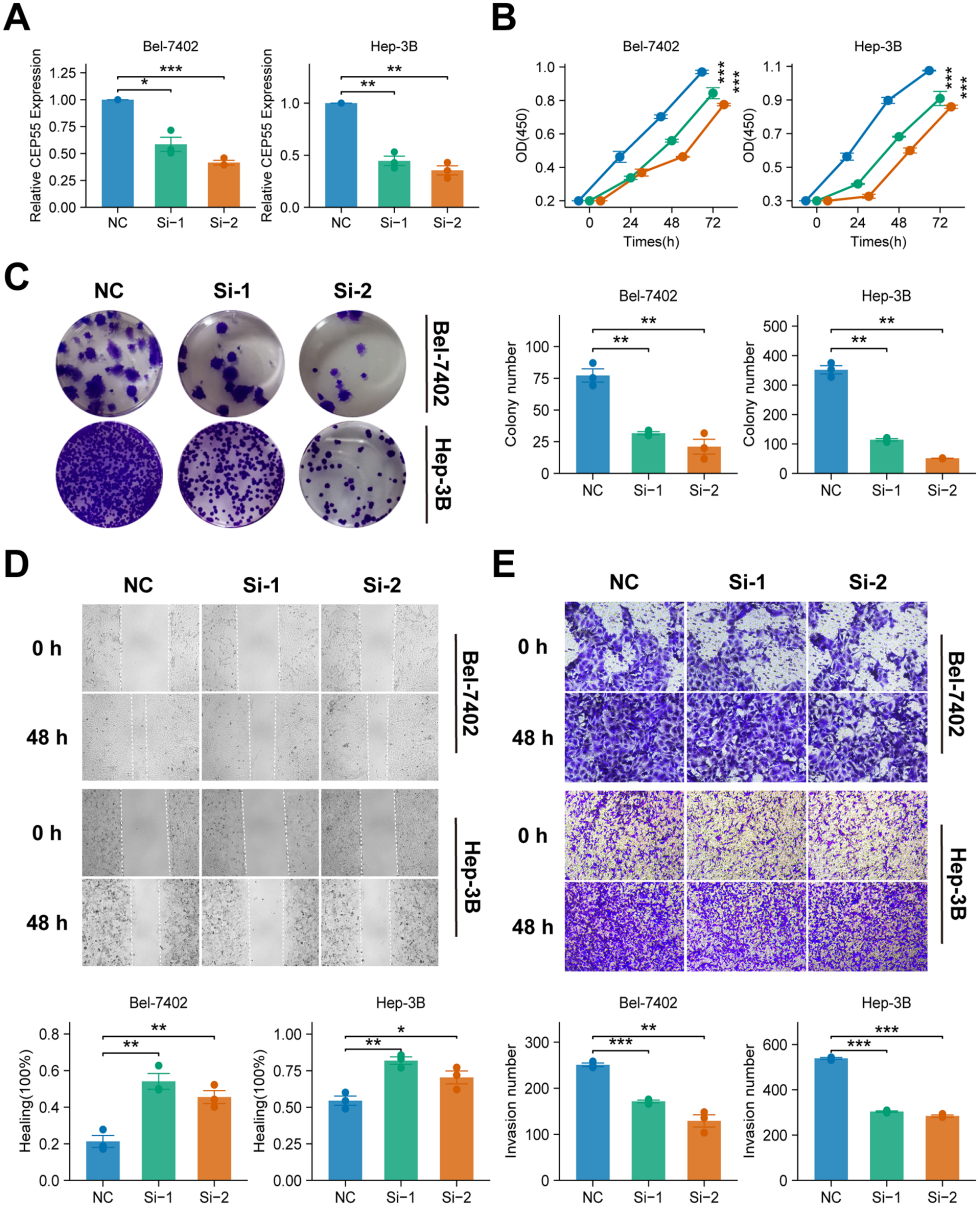
Effects of CEP55 knockdown on cell proliferation, migration and invasion in Bel-7402 and Hep-3B cell lines. **(A)** Quantitative PCR analysis showing the relative expression of CEP55 in Bel-7402 and Hep-3B cells after transfection with siRNA (Si-1 and Si-2) compared to the negative control (NC). **(B)** Cell viability measured at 24, 48, and 72 h in Bel-7402 and Hep-3B cells. Proliferation was significantly reduced in siRNA-treated cells compared to NC. **(C)** Representative images (upper) and quantification (lower) of colony formation in Bel-7402 and Hep-3B cells after CEP55 knockdown. **(D)** Representative images of wound healing at 0 and 48 h (upper) and quantification of healing percentage (lower) in Bel-7402 and Hep-3B cells. **(E)** Representative images (upper) and quantification of invaded cells (lower) in Bel-7402 and Hep-3B cells at 0 and 48 h after CEP55 knockdown. Wilcoxon test was used in A, C, D, **(E)** Chi-square test was used in **(B)** *P < 0.05, **P ≤ 0.01, ***P ≤ 0.001.

In order to validate the critical role of CEP55 in HCC, two different types of CEP55 knockdown phenotypes were constructed for two HCC cell lines, Bel-7402 and Hep-3B. The presence of CEP55 in both HCC cell lines was indicative of the CEP55 knockdown phenotypes. Following the targeting of siRNAs, the gene expression of CEP55 was significantly reduced in cell lines Bel-7402 and Hep-3B (*P < 0.05*) (**Figure 10A**), thus confirming the success of the construction of the CEP55 knockdown phenotypes. Subsequent functional assays revealed that CEP55 knockdown phenotypes Si-1 and Si-2 exhibited diminished cell viability and reduced cell clone number, indicating that CEP55 knockdown inhibited HCC cell proliferation (*P < 0.05*) (**Figures 10B, C**). Furthermore, CEP55 knockdown significantly impeded the migration and invasion of cell lines A and B in the transwell assay and scratch wound healing assay (*P < 0.05*) (**Figures 10D, E**).

### 3.11 Knockdown of CEP55 inhibits tumor growth and proliferation in xenograft models

**Figure 11:**
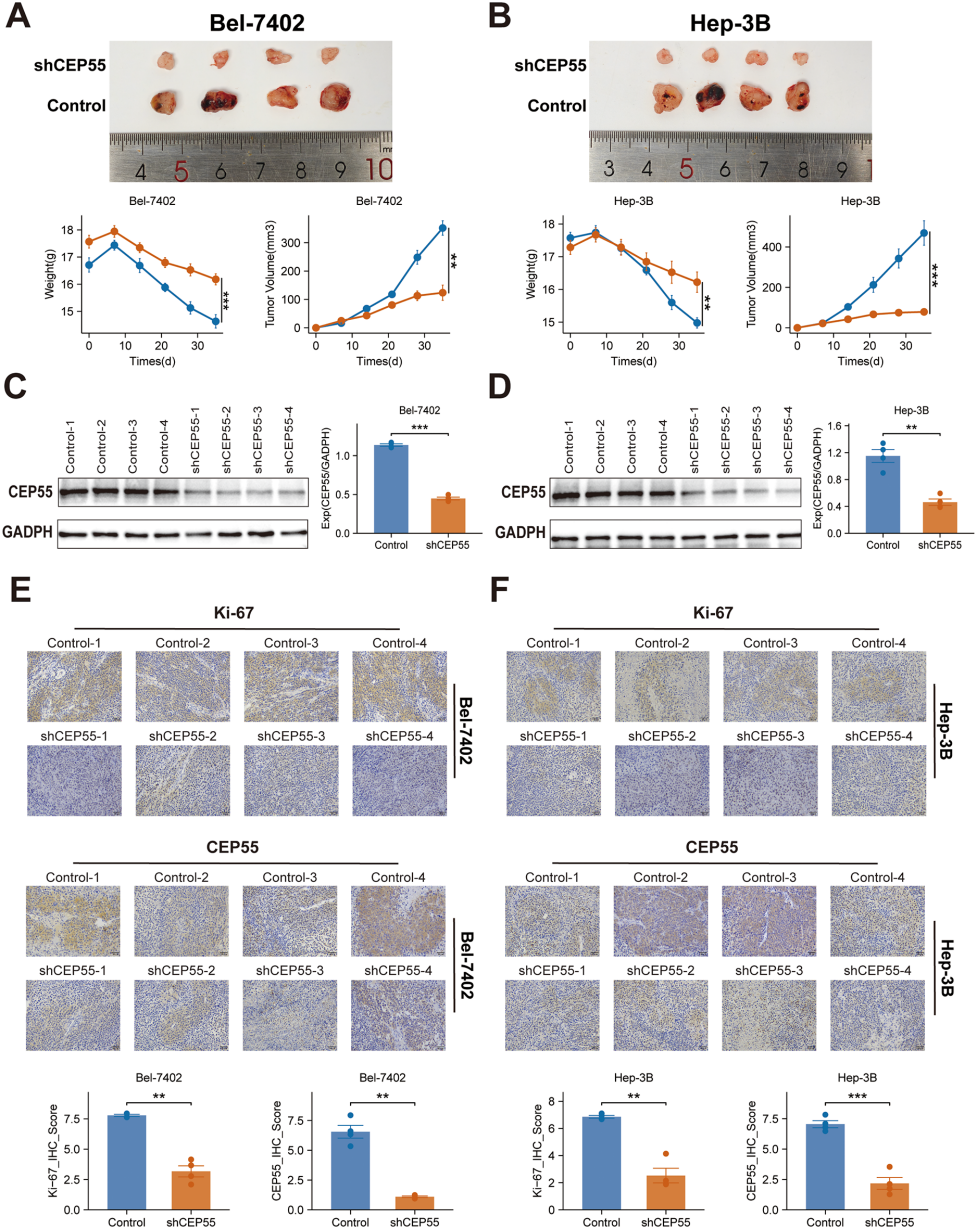
Effects of CEP55 knockdown on tumor growth and proliferation in Bel-7402 and Hep-3B xenograft models. **(A)** Representative images (upper) and quantification (lower) of tumor weight and volume in Bel-7402 xenografts after CEP55 knockdown (shCEP55) and control treatments. **(B)** Representative images (upper) and quantification (lower) of tumor weight and volume in Hep-3B xenografts. **(C)** Western blotting analysis of CEP55 expression in Bel-7402 xenograft tumors. **(D)** Western blotting analysis of CEP55 expression in Hep-3B xenograft tumors. **(E)** Immunohistochemistry staining of Ki-67 and CEP55 in Bel-7402 xenograft tumors (upper) and corresponding quantification of Ki-67 and CEP55 staining (lower). **(F)** Immunohistochemistry staining of Ki-67 and CEP55 in Hep-3B xenograft tumors (upper) and corresponding quantification (lower). *P < 0.05, **P ≤ 0.01, ***P ≤ 0.001..

In order to investigate the effect of CEP55 on tumor growth *in vivo*, xenograft models were established in BALB/c nude mice using Bel-7402 and Hep-3B HCC cell lines with CEP55 knockdown. During the observation period, the activity levels, grooming behavior, and overall health status of the mice remained within normal parameters, exhibiting a downward trend in weight (*P ≤ 0.01*) (**Figures 11A, B**). The body weight of the mice decreased gradually over time, with a more significant decrease observed in the shCEP55 group (*P ≤ 0.01*) (**Figures 11A, B**). Western blotting analysis demonstrated that, in comparison with the control group, the expression levels of CEP55 in the xenograft tumors of the shCEP55 group were significantly reduced, thus indicating that CEP55 knockdown was effective at this particular time (*P ≤ 0.01*) (**Figures 11C, D**). Moreover, CEP55 knockdown effectively inhibited the growth of xenograft tumors derived from Bel-7402 and Hep-3B cells, with tumor volumes in the shCEP55 group being significantly smaller than those in the control group (**Figures 11A, B**). Furthermore, it was found that in xenograft tumors derived from the two different sources, Bel-7402 and Hep-3B, the histochemistry score of CEP55 and Ki-67 in the shCEP55 group were significantly lower than those in the control group, further confirming the inhibitory effect of CEP55 knockdown on tumor cell proliferation (*P ≤ 0.01*) (**Figures 11E, F**).

## 4 Discussion

**Figure 12:**
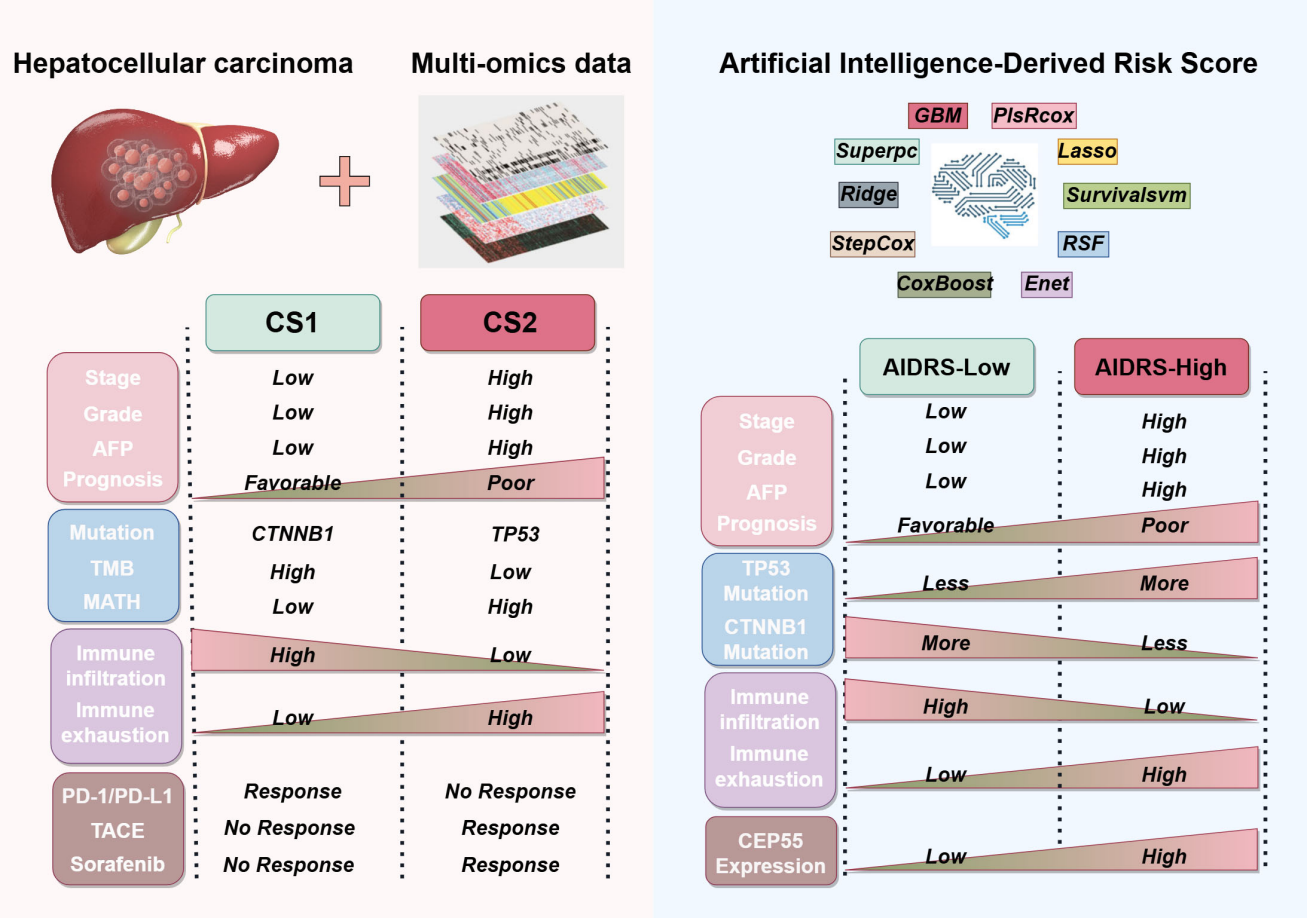
Sketch diagram illustrating the clinicopathological features, genetic alterations, immune status, and treatment responses among CS1 and CS2 based on multi-omics data, as well as the artificial intelligence-derived risk score (AIDRS) classification.

HCC is a highly heterogeneous malignancy, which poses significant challenges to both treatment and prognosis. Despite advancements in systemic therapies, including targeted and immunotherapies, the clinical outcomes for many patients remain suboptimal, particularly in advanced-stage disease. In recent years, multiple molecular subtypes have been proposed for HCC to better understand this heterogeneity and guide personalized treatment. For instance, Hoshida et al. (19) identified three robust HCC subtypes based on transcriptomics: S1, S2, and S3. Among them, S2 has the largest tumor volume and the highest AFP, and has the worst prognosis. In terms of molecular characteristics, S1 exhibits abnormal activation of the WNT signaling pathway, S2 is characterized by cell proliferation, and S3 corresponds to the process of hepatocyte differentiation. In a proteomic study by Guo et al. (20), hepatitis B virus-related HCC patients were divided into metabolic subgroups, microenvironment dysfunction subgroups and proliferation subgroups. S-Mb is characterized by high levels of proteins involved in cancer metabolism and is associated with the best prognosis. In contrast, S-Me is characterized by high levels of proteins involved in immunity and inflammation and is associated with a poorer prognosis when compared with S-Mb. In a systematic study of metabolic gene expression profiles, Chen et al. (45) also identified three subtypes of HCC (C1, C2 and C3). Subtype C1 exhibits high metabolic activity, subtype C2 shows high sensitivity to immunotherapy, and subtype C3 has the highest AFP and the worst prognosis. These efforts have proposed distinct molecular subtypes of HCC and confirmed that these subtypes are associated with treatment response. Nevertheless, studies that focus on a single omics approach or specific biological pathways frequently fail to capture the full complexity of the disease. To address this limitation, our study integrates multi-omics data from genomics, transcriptomics and epigenomics, identifying two molecular subtypes of HCC, CS1 and CS2, with distinct clinical and biological characteristics (**Figure 12**).

In light of the intricacy involved in data integration and the necessity for ensuring the reproducibility of subtypes, the study employed the consensus clustering framework MOVICs (34), a methodology that has been demonstrated to be efficacious in other cancer studies, for the identification of molecular subtypes of HCC. For instance, Ji et al. (46) utilized MOVICs to distinguish between IDH-mutant glioblastoma subtypes, class 1 and class 2, elucidating the disparities in molecular characteristics while identifying drugs, temozolomide and navitoclax, that are sensitive to each of these subtypes. In a similar vein, studies on lung adenocarcinoma (47), colorectal cancer (48), and breast cancer (49) have also employed MOVICs to perform multi-omics typing, thereby enhancing the understanding of tumor heterogeneity and optimizing treatment options for patients. In a manner analogous to these studies, the present study identifies and characterizes HCC subtypes from multiple dimensions, including clinicopathologic features, genetic mutations, DNA methylation patterns, immune microenvironment composition and so on. In this study, we found that CS1 had a more favorable prognosis, while CS2 was associated with poorer clinical outcomes. CS2 was found to have higher serum levels of AFP, longer PT, and larger tumor volume when compared with CS1. As corroborated by both univariate and multivariate Cox regression analysis, underscore the significance of the CS subtype as a critical prognostic factor for patients diagnosed with HCC. The hazard ratio associated with CS1 is notably more effective in predicting outcomes when compared with conventional biomarkers such as AFP, ALB and PT. At the genomic level, CS1 was found to exhibit elevated levels of CTNNB1 mutations and high TMB, while CS2 was predominantly characterized by TP53 mutations and high MATH. It has been demonstrated that gene mutations can induce tumor cells to produce neoantigens by means of regulating gene expression. This, in turn, activates immune cells to recognize and eliminate the tumor cells, thus enhancing the efficacy of immunotherapy (50, 51). In this study, functional enrichment analysis indicated that CS1 with high TMB exhibited significant activation of immune-related pathways, and the infiltration levels of various immune cells, including CD8^+^ T cells, NK cells, and M1 macrophages were significantly higher than those in CS2. Moreover, both computational predictions and real-world data indicated that CS1 exhibited a higher response to immunotherapy. Conversely, Li et al. (52) discovered that TP53 mutations can upregulate MTFHD2 expression to enhance one-carbon metabolism activity in tumor cells, thereby promoting cell proliferation and survival, rendering it an important factor influencing tumor malignant behavior. In a similar vein, it was ascertained that CS2, characterized by a high prevalence of TP53 mutations, exhibited a strong correlation with multiple metabolic pathways. At the same time, Nian et al. (53) demonstrated that TP53 mutations can induce metabolic reprogramming in TAMs in HCC, thereby suppressing the anti-tumor immunity of CD8^+^ T cells. Similarly, it was determined that the CS2 tumor microenvironment is characterized by the predominance of cells, including MDSCs and CAFs, that contribute to immune suppression and immune escape, thereby leading to reduced tumor immunogenicity and immunotherapy response rates. In summary, it is hypothesized that high TMB and high TP53 mutations may be significant driving factors leading to substantial differences in molecular characteristics and clinical outcomes between CS1 and CS2.

As a small molecule inhibitor, nutlin-3 activates the p53 pathway, inducing cell cycle arrest and apoptosis in tumor cells without exerting toxic effects on normal cells (48). It has been demonstrated to possess excellent anti-cancer activity and safety in preclinical studies of retinoblastoma (54), lymphoma (55) and other malignant tumors (56, 57). In this study, we found that CS1 is sensitive to nutlin-3, suggesting that it may benefit significantly from nutlin-3. Conversely, CS1 demonstrated heightened sensitivity to the JAK2 inhibitor ruxolitinib in comparison to CS2. This observation is particularly noteworthy in light of the documented enhanced effect of targeting the JAK2/STAT3 pathway on tumor immunogenicity (58). We hypothesize that this heightened sensitivity is associated with its regulatory influence on the immune microenvironment. Consequently, the combination of ruxolitinib with immune checkpoint inhibitors (ICIs) may yield a more pronounced therapeutic effect for CS1. Conversely, CS2 appears to demonstrate heightened sensitivity to chemotherapeutic agents such as paclitaxel and vinblastine, which are conventional anti-microtubule drugs that have received the Food and Drug Administration (FDA) approval for the treatment of various malignant tumors (59–61), including ovarian cancer, non-small cell lung cancer and breast cancer. However, studies conducted on HCC have thus far been confined to phase II clinical trials (NCT02423239 and NCT04175912). Maybe, the differences between them in drugs sensitivity to CS subtypes may provide a valuable information for subsequent research.

Sorafenib is a first-line treatment for patients with advanced HCC (1). A phase 3 clinical study (5) conducted by the SHARP investigators study group demonstrated that sorafenib can prolong the median survival and time to radiographic progression by nearly 3 months in patients with advanced HCC. However, it is important to note that only a small proportion (20%–40%) of patients with advanced HCC are reported to be sensitive to sorafenib treatment and these patients usually progress after sorafenib treatment (secondary or acquired resistance) (6, 7). For this reason, some special technical methods, such as TACE, are recommended as an important supplementary means of first-line treatment for HCC (62, 63). The current research indicates that the survival outcomes of TACE treatment are variable, with only some patients demonstrating survival benefits (64–66). This study found that patients sensitive to sorafenib and TACE treatment are more concentrated in the CS2 subtype. The tumor microenvironment of CS2 is rich in non-immune cells, such as hepatocytes and fibroblasts, which is undoubtedly optimal for sorafenib and TACE, which exert anti-cancer activity through tumor toxicity. In recent years, there has been vigorous development in research into ICIs for malignant tumors, and related drugs have been widely used in clinical practice (8). The results are not so promising, and some patients have not significantly improved their survival endpoints due to the low response rate (1, 67). However, there is a subset of patients for whom the response to therapy is more favorable, and in whom the immune system can mount a more effective response due to the high immunogenicity of the tumors. This is exemplified by melanoma. By contrast, “cold tumors”, such as those observed in pancreatic cancer and prostate cancer, are characterized by a dense tumor tissue, which hinders the infiltration of immune cells. The immunosuppressive microenvironment of these hinders the effectiveness of immunotherapy. Similarly, CS1 was demonstrated higher immunogenicity due to its abundance of immune cell infiltrations, rendering it more susceptible to immunotherapy.

The application of machine learning (ML) in various fields has led to a growing body of research that substantiates the efficacy of ML technology in predicting disease outcomes, treatment responses, and patient prognoses (68–71). Nevertheless, the selection of the most appropriate model remains challenging due to the heterogeneity of file types, system parameters and dataset formats employed by disparate machine learning algorithms. To address this challenge, this study utilized the Mime framework (72), which integrates 117 distinct machine learning models to construct a prognostic model for HCC patients associated with CS subtypes (72). The “StepCox[forward]+Ent[a=0.1]” model, which demonstrated the highest prediction accuracy, was identified through a comparative analysis of model performance across multiple independent study cohorts. To enhance interpretability and streamline application, artificial intelligence-derived risk score (AIDRS) was further developed based on the model. The findings of this study revealed that elevated AIDRS score were associated with a more unfavorable prognosis for HCC patients with larger tumor volumes, elevated AFP, and longer PT. Six independent study cohorts all confirmed that the AIDRS score for CS2 was significantly higher than that for CS1. Furthermore, the study demonstrated that elevated AIDRS scores are associated with improved immunotherapy efficacy, as evidenced by increased EaSleR and T cell rejection scores, as well as reduced microsatellite instability (MSI) and T cell dysfunction scores. It is evident that AIDRS scores serve a dual purpose; they function as an excellent prognostic risk prediction tool and aid in identifying HCC patient CS subtypes through transcriptomics. This enhances the convenience and universality of subtype classification, thereby guiding personalized treatment for HCC patients. Specifically, patients with higher AIDRS scores tend to belong to CS2. In combination with the elevated response rates documented in CS2 patients to sorafenib or TACE treatment, this substantiates the prioritization of sorafenib or TACE as primary treatment modalities. Conversely, patients exhibiting lower AIDRS scores are more aptly categorized as CS1, signifying a predilection for immunotherapy. Moreover, our findings indicate that CEP55 is a pivotal contributor to AIDRS and associated with unfavorable prognose. In addition, it exhibited an excellent predictive capacity for CS subtypes, with AUC values of 0.942 and 0.822 in the training and validation sets, respectively. Consequently, these findings suggest that CEP55 can serve as a reliable biomarker for distinguishing CS subtypes in HCC patients, which is fully consistent with the AIDRS score. So, the consideration of CEP55 expression may prove advantageous in the development of personalized treatment strategies for HCC patients.

CEP55, a centromere protein, has been shown to be overexpressed in various human cancers, including liver cancer (73), breast cancer (74), and renal cell carcinoma (75). The study (76) found that CEP55 can activate PI3K/AKT and FOXM1-related pathways to intervene in the process of cytokinesis, thereby promoting tumorigenesis, proliferation, and metastasis. In addition, Yang et al. (77) found that patients with HCC overexpressing CEP55 generally had higher histological grades, more lymph node metastases and a poorer prognosis, which is consistent with the findings of the current study. The functional studies of CEP55 in HCC cell lines (Bel-7402 and Hep-3B) demonstrated that CEP55 knockdown inhibited cell proliferation, migration and invasion. Moreover, in xenograft models, CEP55 knockdown significantly reduced tumor growth and proliferation, as evidenced by decreased tumor volume, lower CEP55 and Ki-67 expression. A pan-cancer study by Xie et al. (78) revealed that CEP55 is closely associated with immune-related pathways, such as the IL-6/JAK-STAT3 signaling pathway and the IFN-α/γ response pathway. Additionally, in most malignant tumors, including HCC, CEP55 expression is significantly positively correlated with the infiltration levels of MDSCs and Th2 cells in the tumor microenvironment, leading to immune suppression. In the study, CS2 with high CEP55 expression was found to correspond to higher T cell rejection scores and lower T cell dysfunction scores, overall exhibiting low immunogenicity. On the basis of these findings, it can be speculated that targeting CEP55 may not only directly inhibit tumor cell proliferation and migration but also modulate tumor immunogenicity by influencing immune-related pathways, including the IL-6/JAK-STAT3 and IFN-α/γ pathways, thereby enhancing sensitivity to relevant therapies, particularly immunotherapy.

While the present study provides valuable insights into the molecular subtypes of HCC, there are several limitations that should be acknowledged. Firstly, the retrospective nature of the cohort means that the results may be subject to bias and prospective validation in larger, more diverse patient populations is essential. Secondly, the multi-omics data utilized were limited to genomics, transcriptomics, epigenomics and proteomics. Incorporating additional omics layers, such as metabolomics, could further enhance the predictive power of the models. In addition, while it was demonstrated that the AIDRS had the capacity to predict patient prognosis and guide treatment decisions, its clinical applicability must be confirmed through prospective, multi-central studies. Moreover, further research is required to explore how other therapies, such as combination immune checkpoint inhibitors or novel targeted agents could benefit patients.

## 5 Conclusion

In conclusion, this study successfully identifies and characterizes two distinct HCC subtypes, CS1 and CS2, through the integration of multi-omics data, highlighting their significant differences in clinical outcomes, molecular characteristics and immune features. The development of the AIDRS provides an effective prognostic tool, enabling precise risk stratification and guiding personalized treatment decisions for HCC patients. Of particular note is the identification of CEP55 as a pivotal gene associated with poor prognosis and progression, suggesting that its targeting may offer a promising therapeutic strategy. These findings contribute to a more profound understanding of HCC heterogeneity and lay the foundation for more tailored approaches to treatment, thereby enhancing the precision of clinical interventions in the management of HCC.

## Data Availability

The original contributions presented in the study are included in the article/**Supplementary Material**. Further inquiries can be directed to the corresponding author.

## Glossary

AIDRS: Artificial Intelligence-Derived Risk Score
AFP: Alpha-Fetoprotein
ALB: Albumin
AUC: Area Under the Curve
CCK-8: Cell Counting Kit-8
CIMLR: Cancer Integration via Multikernel Learning
CNV: Copy Number Variation
COCA: Cluster of Clusters Analysis
CPGs: Candidate Prognostic Genes
CPI: Clustering Prediction Index
CS1/CS2: Clustering Subtypes 1 and 2
CTNNB1: Catenin Beta 1
DEGs: Differentially Expressed Genes
DLEU7: Deleted in Lymphocytic Leukemia 7
DMEM: Dulbecco’s Modified Eagle’s Medium
DNA: Deoxyribonucleic Acid
DSS: Disease Specific Survival
EaSIeR: Estimate Systems Immunotherapy Response
EGFR: Epidermal Growth Factor Receptor
Enet: Elastic Network
FBS: Fetal Bovine Serum
FDA: Food and Drug Administration
FPKM: Fragments Per Kilobase of transcript per Million mapped reads
GBM: Generalized Boosted Regression Models
GEO: Gene Expression Omnibus
GISTIC: Genomic Identification of Significant Targets in Cancer
HCC: Hepatocellular Carcinoma
HR: Hazard Ratio
ICGC: International Cancer Genome Consortium
IDH: Isocitrate Dehydrogenase
IFNG: Interferon-Gamma
IntNMF: Integrative Nonnegative Matrix Factorization
JAK2: Janus Kinase 2
Lasso: Least Absolute Shrinkage and Selection Operator
lncRNA: Long Non-Coding RNA
MATH: Mutant-Allele Tumor Heterogeneity
MCPcounter: Microenvironment Cell Populations-counter
MDSC: Myeloid-Derived Suppressor Cells
MET: Mesenchymal-Epithelial Transition
ML: Machine Learning
MOVICS: Multi-Omics integration and Visualization in Cancer Subtyping
MSI: Microsatellite Instability
NC: Negative Control
NCBI: National Center for Biotechnology Information
NF-κB: Nuclear Factor Kappa-Light-Chain-Enhancer of Activated B Cells
NGS: Next-Generation Sequencing
NTP: Nearest Template Prediction
OS: Overall Survival
PBS: Phosphate Buffered Saline
PCs: Principal Components
PECAM1: Platelet and Endothelial Cell Adhesion Molecule 1
PFI: Progression Free Interval
plsRcox: Partial Least Squares Regression for Cox model
PT: Prothrombin Time
qRT-PCR: Quantitative Real-Time Polymerase Chain Reaction
ROC: Receiver Operating Characteristic
RNA-seq: RNA Sequencing
RSF: Random Survival Forest
scRNA-seq: Single-Cell RNA Sequencing
siRNA: Small Interfering RNA
SNP: Single Nucleotide Polymorphism
SNF: Similarity Network Fusion
SPP1: Secreted Phosphoprotein 1
StepCox: Stepwise Cox Regression Model
Superpc: Supervised Principal Components
TACE: Transcatheter Arterial Chemoembolization
TCGA: The Cancer Genome Atlas
TIDE: Tumor Immune Dysfunction and Exclusion
TIMER: Tumor Immune Estimation Resource
TMB: Tumor Mutation Burden
TME: Tumor Microenvironment
TP53: Tumor Protein P53
TTN: Titin
UMAP: Uniform Manifold Approximation and Projection
UMI: Unique Molecular Identifier
VEGF: Vascular Endothelial Growth Factor
VEGFA: Vascular Endothelial Growth Factor A
VWF: Von Willebrand Factor
WNT: Wingless/Integrated Signaling Pathway
