# Supplementary material for: Harnessing multi-omics and artificial intelligence: revolutionizing prognosis and treatment in hepatocellular carcinoma
Source: Front Immunol. 2025 Jul 23;16:1592259. doi: 10.3389/fimmu.2025.1592259 (PMC12325060; doi:10.3389/fimmu.2025.1592259)
Supplement: Supplementary file 2 [file Table1.docx]

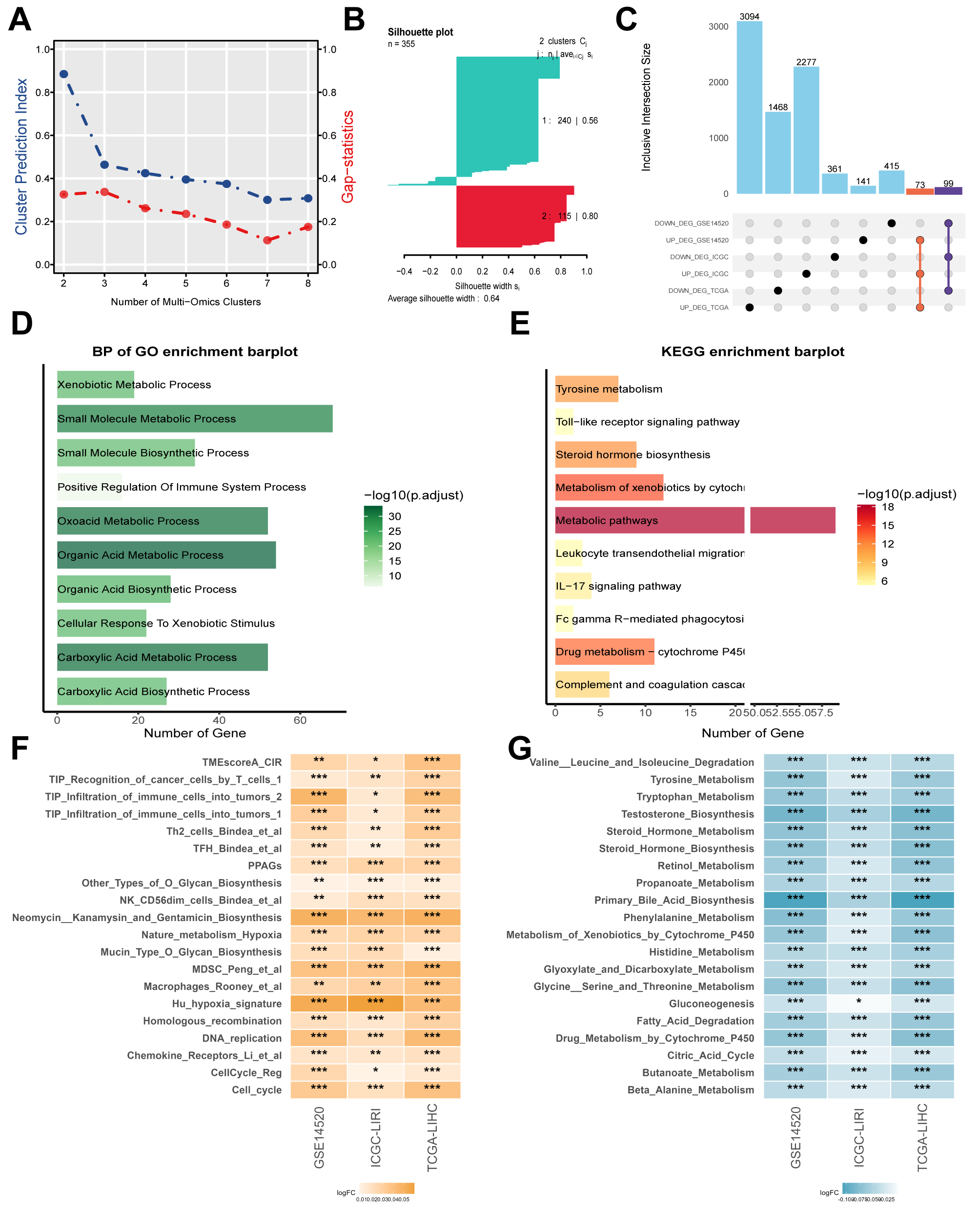


**Supplementary Figure 1.** Multi-omics clustering, differential gene expression analysis and functional enrichment. (A) The cluster prediction index (blue) and gap statistics (red) across varying numbers of multi-omics clusters, indicating the optimal number of clusters at 2. (B) Silhouette analysis comparing the clustering of multi-omics data. (C) Venn diagram and bar chart showing the intersection and size of differentially expressed genes (DEGs) across multiple datasets (TCGA-LIHC, ICGC-LIRI and GSE14520), with gene expression categorized into upregulated and downregulated groups. (D) The GO enrichment barplot of 172 overlapping differentially expressed genes. (E) TheKEGG enrichment barplot of 172 overlapping differentially expressed genes. (F) Heatmap of functional enrichment of across multiple datasets (TCGA-LIHC, ICGC-LIRI and GSE14520). CS2 as control. (F) across multiple datasets (TCGA-LIHC, ICGC-LIRI and GSE14520). CS2 as control. **P* < 0.05, ***P ≤ 0.01*, ****P ≤ 0.001*.


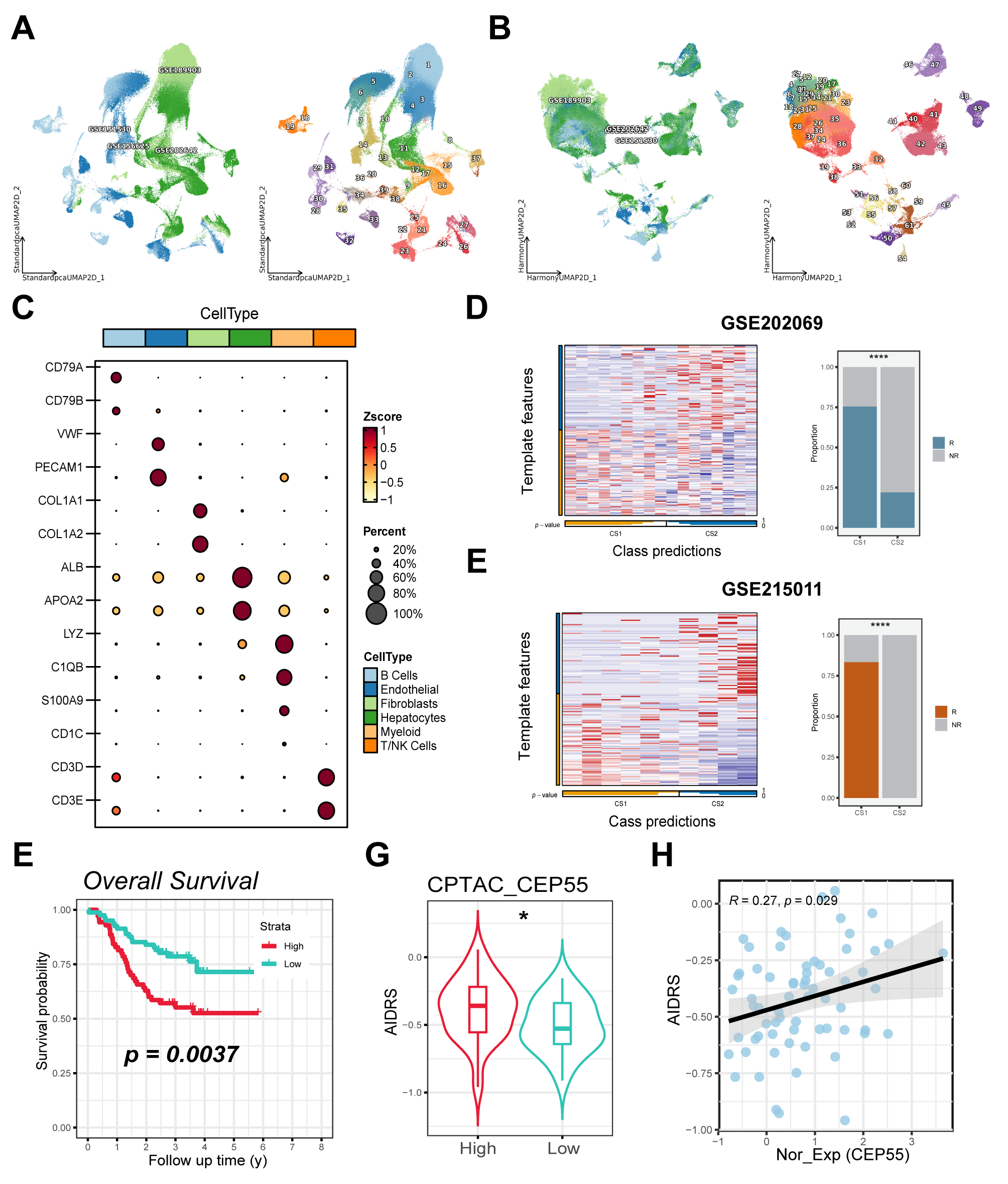


**Supplementary Figure 2.** Dimension reduction, clustering, cellular annotation and external validation. (A) UMAP plot of single-cell RNA-seq data, with distinct clusters identified based on sample data from different cohorts (left) and detailed clusters (right). (B) Integration of single-cell RNA-seq data using harmony, with distinct clusters identified based on sample data from different cohorts (left) and detailed clusters (right). (C) Dot plot showing the expression of key marker genes (CD79A, CD79B, VWF, etc.) across different cell types, with color representing z-scores and circle size indicating the percentage of cells expressing the marker. (D) Evaluation of CS1 and CS2 subtypes in the GSE202069 cohort. (E) Evaluation of CS1 and CS2 subtypes in the GSE215011 cohort. (F) Kaplan-Meier curve corresponding to CEP55 in the CPTAC cohort for overall survival. (G) Violin plots showing AIDRS based on CEP55 across the CPTAC cohort. (H). Correlation between the AIDRS and the normalized expression of CEP55 in the CPTAC cohort. Chi-square test was used in D, E. Log-rank test was used in F. Wilcoxon test was used in G. **P* < 0.05, ***P ≤ 0.01*, ****P ≤ 0.001*.
